# Bioactive Capnosanes and Cembranes from the Soft Coral *Klyxum flaccidum*

**DOI:** 10.3390/md17080461

**Published:** 2019-08-07

**Authors:** Wan-Ru Tseng, Atallah F. Ahmed, Chiung-Yao Huang, Yi-Ying Tsai, Chi-Jen Tai, Raha S. Orfali, Tsong-Long Hwang, Yi-Hsuan Wang, Chang-Feng Dai, Jyh-Horng Sheu

**Affiliations:** 1 Department of Marine Biotechnology and Resources, National Sun Yat-sen University, Kaohsiung 804, Taiwan; 2 Department of Pharmacognosy, College of Pharmacy, King Saud University, Riyadh 11451, Saudi Arabia; 3 Department of Pharmacognosy, Faculty of Pharmacy, Mansoura University, Mansoura 35516, Egypt; 4 Graduate Institute of Natural Products, College of Medicine, Chang Gung University, Taoyuan 333, Taiwan; 5 Research Center for Chinese Herbal Medicine, Research Center for Food and Cosmetic Safety, and Graduate Institute of Health Industry Technology, College of Human Ecology, Chang Gung University of Science and Technology, Taoyuan 333, Taiwan; 6 Department of Anesthesiology, Chang Gung Memorial Hospital, Taoyuan 333, Taiwan; 7 Institute of Oceanography, National Taiwan University, Taipei 112, Taiwan; 8 Frontier Center for Ocean Science and Technology, National Sun Yat-sen University, Kaohsiung 804, Taiwan; 9 Graduate Institute of Natural Products, Kaohsiung Medical University, Kaohsiung 807, Taiwan; 10 Department of Medical Research, China Medical University Hospital, China Medical University, Taichung 404, Taiwan

**Keywords:** soft coral, *Klyxum flaccidum*, capnosane, cembrane, cytotoxic activity, anti-inflammatory activity

## Abstract

Two new capnosane-based diterpenoids, flaccidenol A (**1**) and 7-*epi*-pavidolide D (**2**), two new cembranoids, flaccidodioxide (**3**) and flaccidodiol (**4**), and three known compounds **5** to **7** were characterized from the marine soft coral *Klyxum flaccidum*, collected off the coast of the island of Pratas. The structures of the new compounds were determined by extensive spectroscopic analyses, including 1D and 2D nuclear magnetic resonance (NMR) spectroscopy, and spectroscopic data comparison with related structures. The rare capnosane diterpenoids were isolated herein from the genus *Klyxum* for the first time. The cytotoxicity of compounds **1** to **7** against the proliferation of a limited panel of cancer cell lines was assayed. The isolated diterpenoids also exhibited anti-inflammatory activity through suppression of superoxide anion generation and elastase release in the *N*-formyl-methionyl-leucyl-phenylalanine/cytochalasin B (fMLF/CB)-stimulated human neutrophils. Furthermore, **1** and **7** also exhibited cytotoxicity toward the tested cancer cells, and **7** could effectively inhibit elastase release. It is worth noting that the biological activities of **7** are reported for the first time in this paper.

## 1. Introduction

Chemical investigations on marine invertebrates have disclosed structural diversity in the terpenoid and steroid constituents of octocorals [1]. Cembranoid diterpenes, which contain a 14-membered macro cyclic skeleton, have been shown to exhibit a wide range of biological activities, including cytotoxic [2,3,4,5], antibacterial [6,7,8], anti-inflammatory [3,4,9,10,11], and antiviral [12,13,14] activities. The rarely found cembranoids with 3,7-fused carbobicyclic (capnosane) skeleton have also been found to possess inhibitory effects on protein tyrosine phosphatase 1B, a significant target in treating obesity and type II diabetes [15]; and against A2780 human ovarian tumor cells [16], human disease-related bacteria, and phytopathogens [17]. Additionally, cembranoid-derived diterpenoids are considered to be the main chemical defense of coral against natural predators [18]. Our previous investigation on the chemical constituents of soft corals belonging to the genus *Klyxum* [19,20,21,22,23,24,25,26,27,28,29,30,31,32,33] has shown a wealth of unique secondary metabolites, including cembrane- and eunicellin-based diterpenoids, and steroids. In this context, we isolated a series of cembrane-diterpenoids [32] and steroids [29,31,33] from the Formosan soft corals *Klyxum flaccidum*, some of which have been found to possess cytotoxic and anti-inflammatory activities. A further investigation on the secondary metabolites of the same coral has thus been conducted to discover new metabolites and their bioactivity. In this paper we report the isolation, structure determination, and bioactivity of four new cembrane-derived diterpenoids, flaccidenol A (**1**) and 7-*epi*-pavidolide D (**2**), flaccidodioxide (**3**), and flaccidodiol (**4**), and three known compounds, sarcophytol T (**5**) [34], flaccidoxide-13-acetate (**6**) [35], and 14-*O*-acetylsarcophytol B (**7**) [36]. The structures of compounds were established by extensive spectroscopic analyses (Supplementary Materials, Figures S1 to S28). The cytotoxicities of compounds **1** to **7** were assayed against the cancer cell lines; human lung adenocarcinoma (A549), human colorectal adenocarcinoma (DLD-1), and mouse lymphocytic leukemia (P388D1). Furthermore, the anti-inflammatory activities that occur due to the inhibition of superoxide anion (O_2_^−•^) generation and elastase release in *N*-formyl-methionyl-leucyl-phenylalanine/cytochalasin B (fMLF/CB)-challenged human neutrophils, were evaluated.

## 2. Results and Discussion

The ethyl acetate (EtOAc) extract of *K. flaccidum* was initially fractionated over a silica gel column, and the eluted fractions displaying terpenoidal methyl, olefinic, and oxymethine proton signals in the ^1^H NMR spectra were selected for further purification. One of these fractions was purified by repeated column chromatography to yield diterpenoids **1** to **7** (Figure 1), the structures were established on the basis of spectroscopic analyses (Supplementary Materials, Figures S1 to S28).

**Figure 2:**
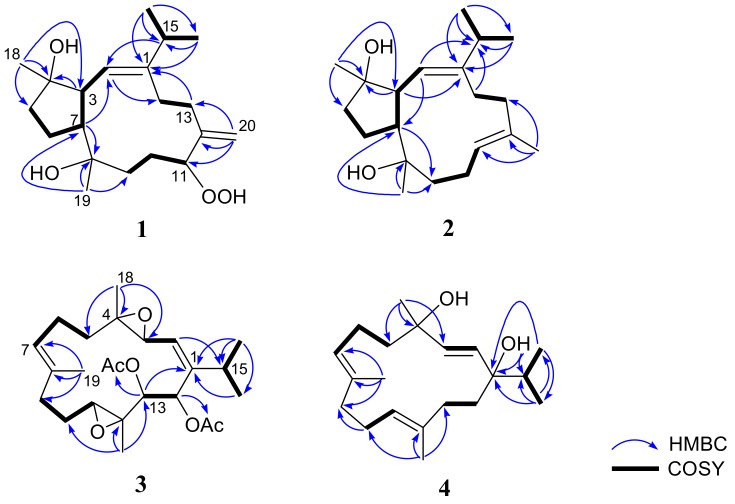
Selected correlation spectroscopy (COSY) and heteronuclear multiple-bond correlation (HMBC) correlations of **1** to **4**.

Flaccidenol A (**1**) was isolated as a colorless oil and has a molecular formula of C_20_H_34_O_4_ with four unsaturations, based on the sodium adduct ion peak [M + Na]^+^ obtained by positive high-resolution electrospray ionization mass spectroscopy (HRESIMS). The infrared (IR) spectrum revealed the presence of hydroxy and olefinic functionalities (ν**_max_** 3391 and 1647 cm^−1^). This was evidenced from the carbon signals of a trisubstituted and one 1,1-disubstituted carbon–carbon double bonds [*δ*_C_ 148.1 (C), 146.6 (C), 122.3 (CH), and 117.4 (CH_2_)] and three *sp^3^* oxygenated carbons [*δ*_C_ 90.7 (CH), 82.8 (C), and 74.5 (C)] (Table 1), respectively, out of the 20 carbon signals in ^13^C NMR spectrum of **1**. Moreover, the remaining two unsaturations indicated that compound **1** was a bicyclic diterpenoid. Similar to cembranoids [32], the ^1^H NMR of **1**, in conjunction with the heteronuclear single quantum coherence (HSQC) spectrum, revealed the presence of six exocyclic carbons of isopropyl [*δ*_H_ 0.97 (3H, d, *J* = 7.0 Hz), 0.90 (3H, d, *J* = 7.0 Hz), and 2.21 (1H, sept, *J* = 7.0 Hz)], two tertiary methyl [*δ*_H_ 1.05 and 0.99 (each 3H, s), and α-methylene [*δ*_H_ 4.95 and 4.93 (each 1H, s)] groups (Table 2). The analysis of correlation spectroscopy (COSY) of **1** indicated four partial structures bearing consecutive proton systems (Figure 2). One of the partial structures included two ring-juncture methines [*δ*_H_/*δ*_C_ 2.89/51.0 (CH) and 2.63/50.5 (CH)] and were positioned at C-3 and C-7 from the analysis of heteronuclear multiple-bond correlation (HMBC) spectra (Figure 2), similar to those of 3,7-cyclized cembranoid (capnosane) diterpenoids [15,17,37,38]. Key HMBC correlations observed from H_3_-18 to C-3 and C-5; H_3_-19 to C-7 and C-9; H_2_-20 to C-11 and C-13; H-15 to C-2 were used to connect the four COSY correlated partial structures and to further establish the 3,7-linkage in the capnosane molecular skeleton. Moreover, the NMR signals at *δ*_H_ 7.64 (1H, br s) and *δ*_C_ 90.7 (CH) pointed out the presence of an allylic hydroperoxy group [39,40,41]. Finally, the detailed HMBC correlation analysis confirmed the hydroperoxyl, the two hydroxyls, the exomethylene, and the trisubstituted double bond to be at C-11, C-4, C-8, C-12, and C-1/C-2, respectively, and consequently the planar structure of **1** as shown in Figure 2.

**Figure 3:**
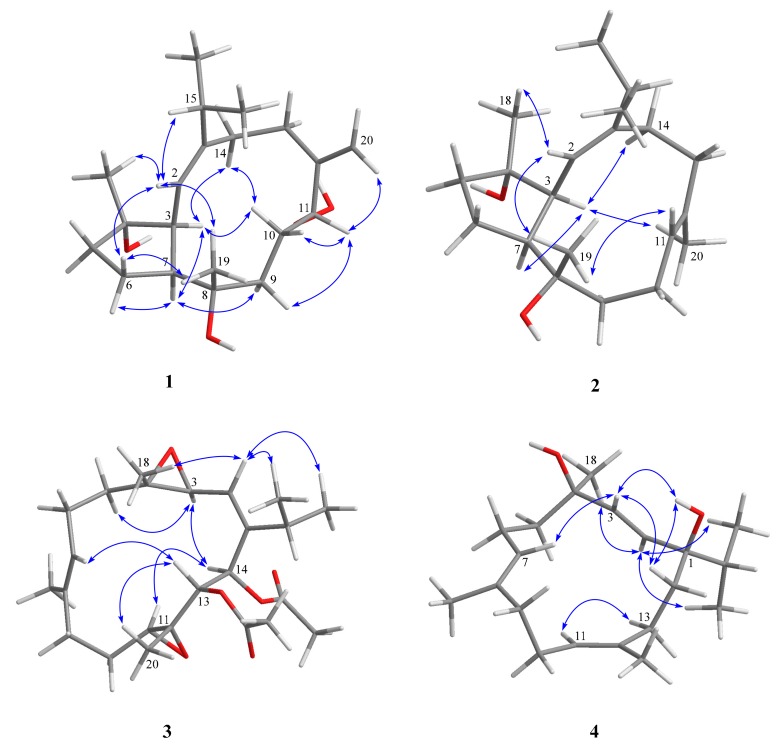
Selected nuclear Overhauser effect (NOE) correlations for **1** to **4**.

The relative configurations of **1** at C-3, C-4, C-7, C-8, and C-11 were proposed from the analysis of nuclear Overhauser effect spectroscopy (NOESY) correlations in combination with molecular modeling using molecular mechanical parameters (MM2 force field) calculations (Figure 3). A strong NOE interaction of H-3 (*δ*_H_ 2.89, dd, *J* = 10.0, 10.0 Hz) with H-7 (*δ*_H_ 2.63, m) indicated the *syn*-orientation of both protons which were arbitrarily assigned as *α*-oriented. The olefinic proton H-2 (*δ*_H_ 4.96, d, *J* = 9.5 Hz) exhibited NOESY correlations with both of H_3_-18 and H_3_-19, but not correlated with H-3. Therefore, H_3_-18 and H_3_-19 should be placed on the β-face of the molecule. One of the methylene protons at C-10 (*δ*_H_ 1.68, ddd, *J* = 13.0, 10.5, 3.0 Hz, H-10α) displayed NOE interaction with H-3, while the other one (*δ*_H_ 1.94, m, H-10β) exhibited NOE correlation with H-11. H-11 is thus β-oriented, and the 11-hydroperoxy group is α-oriented accordingly. The NOE interaction shown by H-11 with H-9β (*δ*_H_ 1.20 ddd, *J* = 13.0, 13.0, 4.5 Hz) and by H-9α (*δ*_H_ 1.85 ddd, *J* = 13.0, 13.0, 3.0 Hz) with H-7 further confirmed the α-orientation of 11-OOH. The *E*-geometry of the 1,2-double bond was assigned from the NOE correlation of H-2 with H-15 of the isopropyl moiety. From the above findings and other detailed NOEs analysis (Figure 3), the relative configuration of **1** was determined as 1*E*, 3S *, 4S *, 7S *, 8S *, 11R *.

The molecular formula of compound **2** was found to be C_20_H_34_O_2_ as deduced from HRESIMS and ^13^C NMR data, appropriate for four degrees of unsaturation. The IR spectrum showed the presence of the hydroxy group (ν**_max_** 3391 cm^−1^) and olefinic functionality (ν**_max_** 1656 cm^−1^). The NMR spectroscopic data were found to be similar to those of pavidolide D [37], while detailed analyses of 2D NMR correlations (COSY and HMBC) revealed that both two compounds possessed the same molecular skeleton (Figure 2). On careful comparison of the ^1^H and ^13^C NMR spectroscopic data (Table 1 and Table 2, respectively) of **2** with those of pavidolide D, it was found that the ring-juncture protons H-3 (*δ*_H_ 2.37, dd, *J* = 9.6, 7.8 Hz) and H-7 (*δ*_H_ 2.65, ddd, *J* = 9.6, 7.8, 6.6 Hz) showed differentiable NMR data with the corresponding H-3 (*δ*_H_ 2.55, t, *J* =10.3 Hz) and H-7 (*δ*_H_ 1.98, m) of the known compound. Further NOE correlations analysis of **2**, confirmed compound **2** to be a new capnosane diterpenoid that should be the 7-epimer of pavidolide D. The assignment of the *cis* ring juncture protons H-3 and H-7 in **2** was further supported by the similar upfield chemical shift C-7 in **2** (*δ*_C_ 54.3) with that of trocheliophol F (*δ*_C_ 54.2) [17] relative to those of the related capnosenes with trans fused rings (*δ*_C_ ≥ 55.5) [16,17,37]. Furthermore, the *E*-geometry of C-11/C-12 double bond was determined from the NOE correlations observed between H_3_-20 and H-3 and between H-11 and H_3_-19, and from the chemical shift of C-20 (δ_C_ < 20 ppm) [42].

Flaccidodioxide (**3**) was obtained as a colorless oil and had a molecular formula of C_24_H_36_O_6_ as established by HRESIMS (m/z 443.2406 [M + Na]^+^, calcd 443.2404), corresponding to seven degrees of unsaturation. IR absorption at 1746 cm^−1^ showed the presence of ester carbonyl group. Comparison of the NMR spectroscopic data of **3** (Table 1 and Table 2), with those of a known metabolite gibberosene C [43], revealed that both compounds possessed the same 3(4),11(12)-diepoxyl groups in the cembrane ring-structure. The hydroxyl group at C-13 of gibbrosene C was acetylated, and the methylene group of C-14 was converted to acetoxyl-bearing methine, as support by the downfield chemical shifts of H-13 (*δ*_H_ 5.49) and H-14 (*δ*_H_ 5.89) of **3**, and the three-bond correlations of both protons to two acetate carbonyl carbons unveiled from HMBC spectrum of **3**. The planar structure of **3** was further confirmed by the detailed analyses of ^1^H-^1^H COSY and HMBC spectra (Figure 2). These results, along with extensive analysis of NOE correlations of **3** (Figure 3), determined the structure of **3** unambiguously.

Flaccidodiol (**4**) was isolated as a gum. HRESIMS of **4** exhibited a sodiated molecular ion peak at *m*/*z* 329.2448, corresponding to the molecular formula C_20_H_34_O_2_. IR absorptions at 3457 cm^−1^ indicated the presence of a hydroxyl group in **4**. The NMR data (Table 1 and Table 2) assigned the presence of an isopropyl group and three methyls, including two olefinic ones and one attaching to an oxygenated *sp^3^* carbon atom. It was found that the proton and carbon chemical shifts of **4** were very similar to those of a known dihydroxycembrane, sarcophytol T [34]. Thus, both compounds should be diastereomers. Furthermore, the proton signal of 1-OH exhibited NOE correlation with H-3, while H_3_-18 showed NOE correlation with H-2, revealing the position of both 1-OH and 4-OH to be in the same face of **4**. Thus, the relative configuration for **4** was established.

Cytotoxicities of metabolites **1** to **7** against the growth of human lung adenocarcinoma (A549), human colorectal adenocarcinoma (DLD-1), and mouse lymphocytic leukemia (P388D1) cell lines were screened. Compounds **1**, **2,** and **7** exhibited inhibitory activity against the growth of the tested cancer cells (Table 3). The hydroperoxyl (as in **1**) seem to potentiate the cytotoxic effect of the diterpenoid molecules. However, the diepoxide compound **3** could selectively inhibit the growth of P388D1 cancer cells relative to the inactive monoepoxide derivative (**6**). These compounds also exhibited the potent anti-inflammatory activity by suppressing O_2_^−•^ generation and elastase release in the fMLF/CB-stimulated human neutrophils (Table 4). Compound **7** could effectively inhibit elastase release (IC_50_ = 7.22 ± 0.85 µM), reducing the level of elastase release to 59.66 ± 0.83% at a concentration of 10 µM relative to the control group.

## 3. Experimental Section

### 3.1. General Experimental Procedures

Optical rotations and IR spectra were measured on a JASCO P-1020 polarimeter and JASCO FT/IR-4100 spectrophotometer (JASCO Corporation, Tokyo, Japan), respectively. NMR spectra were recorded on a Varian 400 FT-NMR (Bruker, Bremen, Germany) at 400 and 100 MHz for ^1^H and ^13^C, respectively; and on a Varian Unity INOVA 500 FT-NMR (Varian Inc., Palo Alto, CA, USA) at 500 and 125 MHz for ^1^H and ^13^C, respectively, in C_6_D_6_ or CDCl_3_ (*δ* in ppm, *J* in Hz). Silica and RP-18 silica gels (230 to 400 mesh), and precoated silica gel 60 F-254 (0.2 mm) plates (Merck, Darmstadt, Germany) were used for column and thin-layer chromatography, respectively. Preparative high-performance liquid chromatography (HPLC) was performed on a Hitachi L-2455 HPLC apparatus (Hitachi Ltd., Tokyo, Japan) with a Supelco C18 (250 × 21.2 mm, 5 μm) column (Supelco, Bellefonte, PA, USA).

### 3.2. Animal Material

The soft coral *Klyxum flaccidum* Tixier-Durivault was collected along the coast of Pratas island, Taiwan, and stored until extraction as described before [32]. The organism was identified by one of the co-authors (C.-F.D.). A voucher sample (No. LI6) was deposited at the Department of Marine Biotechnology and Resources, National Sun Yat-sen University.

### 3.3. Extraction and Isolation

The frozen bodies of *K. flaccidum* (8.0 kg, wet weight) were sliced and worked up as described previously to yield EtOAc extract (120 g) [32]. Twenty-six fractions were eluted by silica gel column chromatography, using *n*-hexane, EtOAc–*n*-hexane (0:100 to 100:0, gradient), and subsequently MeOH–EtOAc (1:100 to 100:1, gradient). Based on the analysis of ^1^H NMR spectra, the terpenoids-containing fractions were selected for further chromatographic separation. Fraction **7** eluted with EtOAc–*n*-hexane (1:3) was purified by a Sephadex LH-20 column using acetone as eluent to obtain a mixture (**7a** to **7c**), Subfraction **7a** was further separated by reversed-phase RP-18 silica gel column using MeOH–H_2_O (3:1) to give **3** (4.8 mg) and **5** (34.6 mg), and subfraction **7c** using MeOH–H_2_O (4:1) to give **4** (1.3 mg). Subfraction **7b** was further separated by RP-18 silica gel column using MeOH–H_2_O (4:1), and further semipreparative RP-18 HPLC (MeOH–H_2_O, 3:1) to yield compounds **6** (3.4 mg) and **7** (2.1 mg). Fraction 13, eluted with EtOAc–*n*-hexane (3:1), was also rechromatographed by a Sephadex LH-20 column, using acetone as the mobile phase, and then was further purified by RP-18 HPLC using MeOH–H_2_O (5:1) to afford **1** (1.5 mg) and **2** (9.7 mg).

Flaccidenol A (**1**): Colorless oil; ${\left[\alpha \right]}_{D}^{25}$ −10.3 (*c* 0.43, CHCl_3_); IR (neat) ν_max_ 3391, 2957, 2870, 1647, 1455, 1376, 1109, 912, 754, and 614 cm^–1^; ^13^C NMR data (125 MHz; C_6_D_6_), see Table 1; ^1^H NMR data (500 MHz; C_6_D_6_), see Table 2; ESIMS *m*/*z* 361 [M + Na]^+^; HRESIMS *m*/*z* 361.2349 [M + Na]^+^ (calcd for C_2__0_H_34_O_4_Na, 361.2349).

7-*epi*-Pavidolide D (**2**): Colorless oil; ${\left[\alpha \right]}_{D}^{25}$ −3.8 (*c* 2.77, CHCl_3_); IR (neat) ν_max_ 3391, 2957, 2865, 1656, 1451, 1372, 1127, 908, and 754 cm^–1^; ^13^C NMR data (100 MHz; CDCl_3_), see Table 1; ^1^H NMR data (400 MHz; CDCl_3_), see Table 2; ESIMS *m*/*z* 329 [M + Na]^+^; HRESIMS *m*/*z* 329.2453 [M + Na]^+^ (calcd for C_20_H_34_O_2_Na, 329.2452).

Flaccidodioxide (**3**): Colorless oil; ${\left[\alpha \right]}_{D}^{25}$ +13.0 (*c* 0.50, CHCl_3_); IR (neat) ν**_max_** 2962, 2930, 2872, 1746, 1455, 1373, 1223, 1029, 966, 877, and 757 cm^–1^; ^13^C NMR data (100 MHz; CDCl_3_), see Table 1; ^1^H NMR data (400 MHz; CDCl_3_), see Table 2; ESIMS *m*/*z* 443 [M + Na]^+^; HRESIMS *m*/*z* 443.2406 [M + Na]^+^ (calcd for C_24_H_36_O_6_Na, 443.2404).

Flaccidodiol (**4**): Colorless oil; ${\left[\alpha \right]}_{D}^{25}$ −65.0 (*c* 0.25, CHCl_3_); IR (neat) ν**_max_**3457, 2925, 1450, 1368, 1095, and 986 cm^–1^; ^13^C NMR data (125 MHz; CDCl_3_), see Table 1; ^1^H NMR data (500 MHz; CDCl_3_), see Table 2; ESIMS *m*/*z* 329 [M + Na]^+^; HRESIMS *m*/*z* 329.2448 [M + Na]^+^ (calcd for C_20_H_34_O_2_Na, 329.2451).

### 3.4. Cytotoxicity Assay

The cancer cell lines were purchased from American Type Culture Collection (ATCC). Cytotoxicity of the compounds were measured using 3-(4,5-dimethylthiazol-2-yl)-2,5-diphenyltetrazolium bromide (MTT) colorimetric method [44,45]. The positive control used was doxorubicin. The compound was considered inactive if IC_50_ > 20 μg/mL.

### 3.5. In Vitro Anti-Inflammatory Assay

Human neutrophils were obtained from blood by dextran sedimentation, Ficoll-Hypaque centrifugation, and hypotonic lysis and then incubated as previously described [46]. Neutrophils (6 × 10^5^ cells mL^−1^) incubated in Hank’s Balanced Salt Solution (HBSS) with MeO-Suc-Ala-Ala-Pro-Val-*p*-nitroanilide (100 μM), and Ca^2+^ (1 mM) at 37 °C were treated with the tested compound or Dimethyl sulfoxide (DMSO) for 5 min. The activation of neutrophils was challenged for 10 min with fMLF (100 nM)/CB (0.6 and 0.5 μg/mL^‒1^ for O_2_^−•^ generation and elastase release, respectively). The anti-inflammatory activity of compounds **2** to **7** were assessed by measuring the inhibition of fMLF/CB-stimulated cells producing O_2_^−•^ and elastase by using UV spectrometer detection at wavelength 550 nm and 405 nm, respectively [46,47].

## 4. Conclusions

Further chemical investigation on Formosan soft coral *Klyxum flaccidum* has led to the isolation and characterization of two new capnosanoids, flaccidenols A (**1**) and B (**2**); two new cembranoids, flaccidodioxide (**3**) and flaccidodiol (**4**); along with three known cembranoids. This is the first study to discover the rarely found capnosane diterpenoids from genus *Klyxum*. Diterpenoids **1**, **2**, and **7** exhibited significant cytotoxicity against a limited panel of cancer cell lines. The hydroperoxy group might be required to potentiate the cytotoxic effect, as shown by the capnosanoid **1**. Moreover, 14-*O*-acetylsarcophytol B (**7**) could inhibit the elastase release in the fMLF/CB-stimulated human neutrophils effectively. This is the first time to disclose bioactivity for this known compound, which might be considered as an anti-inflammatory candidate.

## Figures and Tables

**Figure 1:**
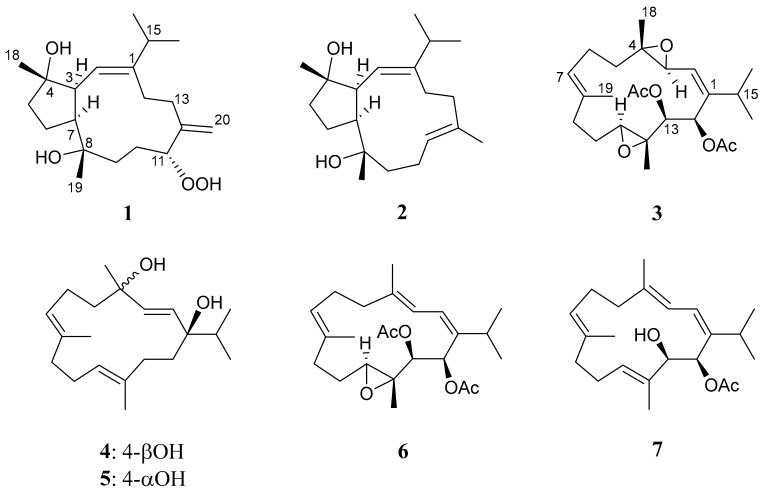
Structures of compounds **1** to **7**.

**Table 1:** ^13^C nuclear magnetic resonance (NMR) data of compounds **1** to **4**.

Position	1 *^a^*	2 *^b^*	3 *^b^*	4 *^c^*
1	146.6, C	144.4, C	149.6, C	78.6, C
2	122.3, CH *^d^*	121.8, CH	124.8, CH	131.5, CH
3	51.0, CH	51.1, CH	58.1, CH	136.5, CH
4	82.8, C	82.8, C	62.1, C	72.3, C
5	40.3, CH_2_	37.7, CH_2_	37.8, CH_2_	43.9, CH_2_
6	25.5, CH_2_	23.0, CH_2_	23.5, CH_2_	22.3, CH_2_
7	50.5, CH	54.3, CH	126.4, CH	128.4, CH
8	74.5, C	75.2, C	133.7, C	132.8, C
9	37.9, CH_2_	44.1, CH_2_	35.9, CH_2_	39.0, CH_2_
10	25.4, CH_2_	23.1, CH_2_	24.0, CH_2_	23.9, CH_2_
11	90.7, CH	127.1, CH	59.1, CH	126.5, CH
12	148.1, C	132.3, C	60.4, C	136.3, C
13	30.3, CH_2_	36.0, CH_2_	73.2, CH	36.2, CH_2_
14	28.6, CH_2_	28.3, CH_2_	67.8, CH	32.7, CH_2_
15	32.7, CH	32.9, CH	28.9, CH	38.7, CH
16	21.1, CH_3_	21.2, CH_3_	24.2, CH_3_	16.7, CH_3_
17	23.7, CH_3_	23.8, CH_3_	24.0, CH_3_	17.6, CH_3_
18	25.8, CH_3_	25.2, CH_3_	17.5, CH_3_	27.8, CH_3_
19	27.1, CH_3_	22.1, CH_3_	15.9, CH_3_	14.8, CH_3_
20	117.4, CH_2_	17.3, CH_3_	16.2, CH_3_	14.7, CH_3_
13-OAc			20.7, CH_3_	
			170.4, C	
14-OAc			20.7, CH_3_	
			169.0, C	

*^a^* Spectrum recorded in C_6_D_6_ at 125 MHz at 25 °C, *^b^* Spectra recorded in CDCl_3_ at 100 MHz at 25 °C, *^c^* Spectrum recorded in CDCl_3_ at 125 MHz at 25 °C, *^d^* Attached protons were determined by distortionless enhancement by polarization transfer (DEPT) experiments.

**Table 2:** ^1^H NMR spectral data for compounds **1** to **4**.

Position	1 *^a^*	2 *^b^*	3 *^b^*	4 *^c^*
2	4.96 d (9.5) *^d^*	5.05 d (10.8)	5.25 m	5.59 d (16.0)
3	2.89 dd (10.0, 10.0)	2.37 dd (7.8, 9.6)	3.56 d (9.6)	6.05 d (16.0)
5	1.52 2H, m	1.72 m; 1.65 m	1.48 m; 2.11 m	1.52 m; 1.99 m
6	1.94 m; 1.64 m	1.88 m; 1.76 m	2.13 m; 2.23 m	2.22 m; 2.35 m
7	2.63 m	2.65 ddd (6.6, 7.8, 9.6)	5.22 m	5.34 dd (7.5, 7.5)
9	1.85 ddd (13.0, 13.0, 3.0); 1.20 ddd (13.0, 13.0, 4.5)	1.88 m; 2.00 m	2.14 m; 2.24 m	1.96 m; 2.18 m
10	1.94 m; 1.68 ddd (13.0, 10.5, 3.0)	2.14 m	1.09 m; 1.61 m	2.04 m; 2.23 m
11	4.27 dd (11.0, 4.5)	4.99 dd (7.6, 7.6)	3.12 dd (5.6, 5.6)	5.18 dd (9.0, 3.5)
13	2.66 m; 2.20 dd (11.5, 5)	2.03 m; 1.92 m	5.49 d (7.6)	2.10 m; 2.15 d (3.0)
14	2.79 ddd (11.5, 11.5, 4.5); 2.00 m	2.40 dd (14.4, 7.2); 1.94 ddd (14.4, 12.0, 3.0)	5.89 d (7.6)	1.86 td (12.5, 3.5); 1.60 m
15	2.22 sept (7.0)			
16	0.97 3 H, d (7.0)	1.06 3H, d (7.2)	1.02 3H, d (6.4)	0.87 3H, d (7.0)
17	0.90 3 H, d (7.0)	1.02 3H, d (6.8)	1.02 3H, d (6.4)	0.87 3H, d (7.0)
18	0.99 3 H, s	1.14 3H, s	1.33 3H, s	1.40 3H, s
19	1.05 3 H, s	1.25 3H, s	1.64 3H, s	1.60 3H, s
20	4.93 s; 4.97 s	1.67 3H, s	1.26 3H, s	1.67 3H, s
13-OAc			2.14 3H, s	
14-OAc			2.01 3H, s	
1-OH				2.39 s
11-OOH	7.64 br s			

*^a^* Spectrum recorded in C_6_D_6_ at 500 MHz at 25 °C, *^b^* Spectra recorded in CDCl_3_ at 400 MHz at 25 °C, *^c^* Spectrum recorded in CDCl_3_ at 500 MHz at 25 °C, *^d^ J* values (Hz) in parentheses.

**Table 3:** Cytotoxicity (IC_50_ μg/mL) of compounds **1** to **7**.

Compound	Cell Lines IC_50_ (μg/mL)		
	A549 *^a^*	DLD-1 *^b^*	P388D1 *^c^*
**1**	9.7 ± 1.2	6.0 ± 0.4	7.2 ± 1.8
**2**	28.6 ± 3.8	31.6 ± 3.7	30.4 ± 4.8
**3**	− *^d^*	−	19.6 ± 8.3
**4**	−	−	−
**5**	−	−	−
**6**	−	−	−
**7**	10.8 ± 4.9	11.7 ± 4.8	8.9 ± 2.2
Doxorubicin *^e^*	0.3 ± 0.1	1.5 ± 0.2	0.9 ± 0.2

*^a^* A549: Human lung adenocarcinoma. *^b^* DLD-1: Human colorectal adenocarcinoma. *^c^* P388D1: Mouse lymphocytic leukemia cell line. *^d^* IC_50_ > 40 μg/mL. *^e^* Clinical anticancer drug used as a positive control.

**Table 4:** Inhibitory effects of compounds **2** to **7** on superoxide anion generation and elastase release in *N*-formyl-methionyl-leucyl-phenylalanine/cytochalasin B (fMLF/CB)-induced human neutrophils at 10 μM.

Compounds	Superoxide Anion			Elastase Release		
	IC_50_ (μM) *^a^*	Inh % *^b^*		IC_50_ (μM) *^a^*	Inh % *^b^*	
**2**	>10	24.46 ± 6.99	*	>10	29.96 ± 6.14	**
**3**	>10	8.88 ± 3.33		>10	27.18 ± 4.05	**
**4**	>10	3.29 ± 0.88	*	>10	14.43 ± 3.75	*
**5**	>10	8.89 ± 4.28		>10	14.89 ± 3.62	*
**6**	>10	4.73 ± 1.53	*	>10	3.09 ± 3.88	
**7**	>10	11.95 ± 2.53	**	7.22 ± 0.85	59.66 ± 0.83	***
**Idelalisib**	0.07 ± 0.01	102.8 ± 2.2	***	0.3 ± 0.1	99.6 ± 4.2	***

*^a^* Concentration necessary for 50% inhibition (IC_50_). *^b^* Percentage of inhibition (Inh %) at 10 μM. Results are shown as mean ± S.E.M. (*n* = 3–4). * *p* < 0.05, ** *p* < 0.01, *** *p* < 0.001 compared with the control value.

## Supplementary Materials

HRESIMS and NMR spectra of new compounds **1** to **4** are available online at https://www.mdpi.com/1660-3397/17/8/461/s1. Figure S1: HRESIMS spectrum of **1**, Figure S2: ^1^H NMR spectrum of **1** in C_6_D_6_ at 500 MHz, Figure S3: ^13^C NMR spectrum of **1** in C_6_D_6_ at 125 MHz, Figure S4: ^1^H-^1^H COSY spectrum of **1** in C**6**D**6**, Figure S5: HSQC spectrum of **1** in C_6_D_6_, Figure S6: HMBC spectrum of **1** in C_6_D_6_, Figure S7: NOESY spectrum of **1** in C_6_D_6_, Figure S8: HRESIMS spectrum of **2**, Figure S9: ^1^H NMR spectrum of **2** in CDCl_3_ at 400 MHz, Figure S10: ^13^C NMR spectrum of **2** in CDCl_3_ at 100 MHz, Figure S11: ^1^H-^1^H COSY spectrum of **2** in CDCl_3_, Figure S12: HSQC spectrum of **2** in CDCl_3_, Figure S13: HMBC spectrum of **2** in CDCl_3_, Figure S14: NOESY spectrum of **2** in CDCl_3_, Figure S15: HRESIMS spectrum of **3**, Figure S16: ^1^H NMR spectrum of **3** in CDCl_3_ at 400 MHz, Figure S17: ^13^C NMR spectrum of **3** in CDCl_3_ at 100 MHz, Figure S18: ^1^H-^1^H COSY spectrum of **3** in CDCl_3_, Figure S19: HSQC spectrum of **3** in CDCl_3_, Figure S20: HMBC spectrum of **3** in CDCl_3_, Figure S21: NOESY spectrum of **3** in CDCl_3_, Figure S22: HRESIMS spectrum of **4**, Figure S23: ^1^H NMR spectrum of **4** in CDCl_3_ at 500 MHz, Figure S24: ^13^C NMR spectrum of **4** in CDCl_3_ at 125 MHz, Figure S25: ^1^H-^1^H COSY spectrum of **4** in CDCl_3_, Figure S26: HSQC spectrum of **4** in CDCl_3_, Figure S27: HMBC spectrum of **3** in CDCl_3_, Figure S28: NOESY spectrum of **3** in CDCl_3_.
