# Supplementary material for: Bioactive Capnosanes and Cembranes from the Soft Coral Klyxum flaccidum
Source: Mar Drugs. 2019 Aug 7;17(8):461. doi: 10.3390/md17080461 (PMC6722650; doi:10.3390/md17080461)
Supplement: Supplementary file 1 [file marinedrugs-17-00461-s001.pdf]

Bioactive Capnosanes and Cembranes from the Soft Coral Klyxum Flaccidum

Wan-Ru Tseng, Atallah F. Ahmed, Chiung-Yao Huang, Yi-Ying Tsai, Chi-Jen Tai, Raha S. Orfali, Tsong-Long Hwang, Yi-Hsuan Wang, Chang-Feng Dai and Jyh-Horng Sheu *

List of Supplementary material

No.	Content	Page
**Figure S1**	HRESIMS spectrum of **1**	S2
**Figure S2**	^{1}H NMR spectrum of **1** in C_6D_6 at 500 MHz	S3
**Figure S3**	^{13}C NMR spectrum of **1** in C_6D_6 at 125 MHz	S4
**Figure S4**	^{1}H–^{1}H COSY spectrum of **1** in C_6D_6	S5
**Figure S5**	HSQC spectrum of **1** in in C_6D_6	S6
**Figure S6**	HMBC spectrum of **1** in in C_6D_6	S7
**Figure S7**	NOESY spectrum of **1** in in C_6D_6	S8
**Figure S8**	HRESIMS spectrum of **2**	S9
**Figure S9**	^{1}H NMR spectrum of **2** in $CDCl_3$ at 400 MHz	S10
**Figure S10**	^{13}C NMR spectrum of **2** in $CDCl_3$ at 100 MHz	S11
**Figure S12**	HSQC spectrum of **2** in in $CDCl_3$	S12
**Figure S13**	HMBC spectrum of **2** in in $CDCl_3$	S13
**Figure S14**	NOESY spectrum of **2** in in $CDCl_3$	S14
**Figure S15**	HRESIMS spectrum of **3**	S15
**Figure S16**	^{1}H NMR spectrum of **3** in $CDCl_3$ at 400 MHz	S16
**Figure S17**	^{13}C NMR spectrum of **3** in $CDCl_3$ at 100 MHz	S17
**Figure S18**	^{1}H–^{1}H COSY spectrum of **3** in $CDCl_3$	S18
**Figure S19**	HSQC spectrum of **3** in in $CDCl_3$	S19
**Figure S20**	HMBC spectrum of **3** in in $CDCl_3$	S20
**Figure S21**	NOESY spectrum of **3** in in $CDCl_3$	S21
**Figure S22**	HRESIMS spectrum of **4**	S22
**Figure S23**	^{1}H NMR spectrum of **4** in $CDCl_3$ at 500 MHz	S23
**Figure S24**	^{13}C NMR spectrum of **4** in $CDCl_3$ at 125 MHz	S24
**Figure S25**	^{1}H–^{1}H COSY spectrum of **4** in $CDCl_3$	S25
**Figure S26**	HSQC spectrum of **4** in in $CDCl_3$	S26
**Figure S27**	HMBC spectrum of **4** in in $CDCl_3$	S27
**Figure S28**	NOESY spectrum of **4** in in $CDCl_3$	S28

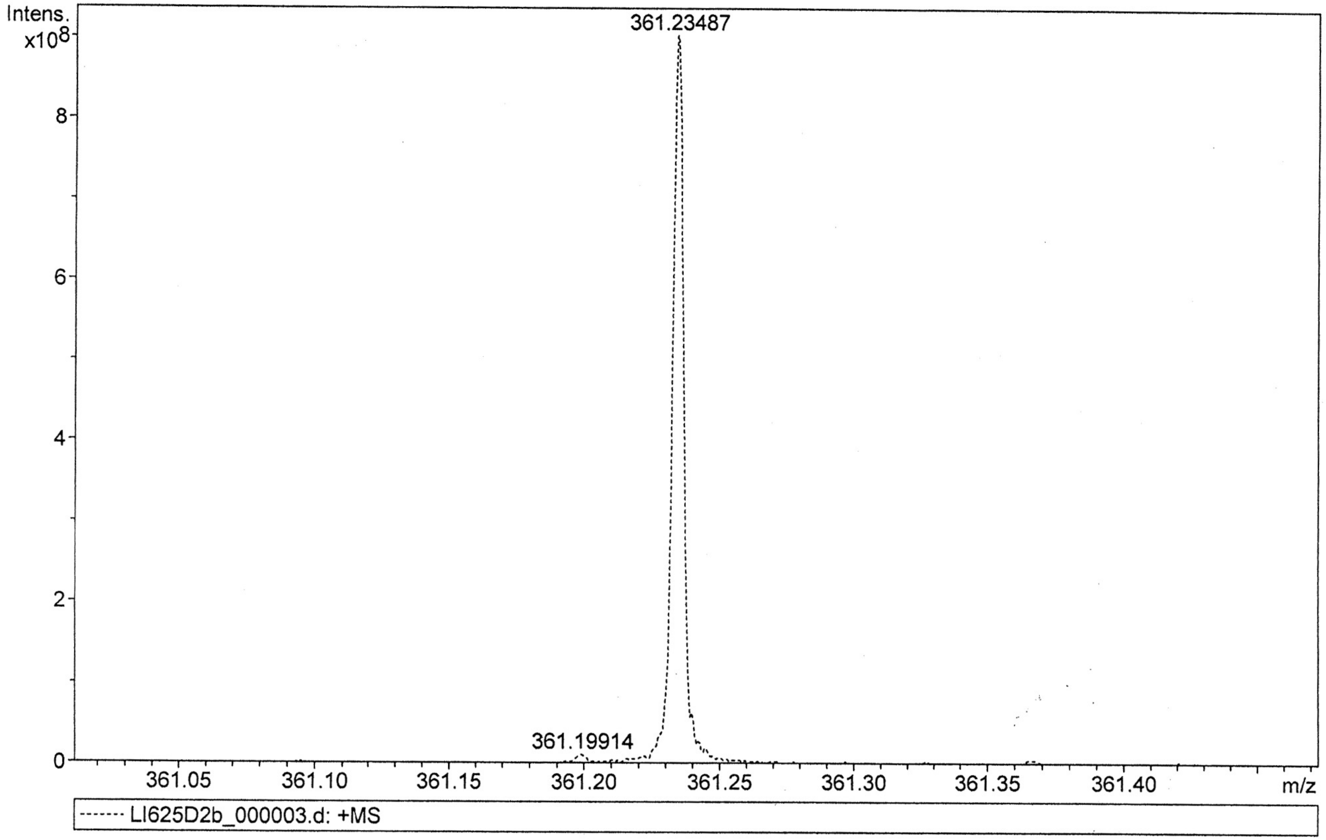

Meas. m/z	#	Formula	Score	m/z	err [mDa]	err [ppm]	mSigma	rdb	e⁻ Conf	N-Rule
361.23487	1	C 20 H 34 Na O 4	100.00	361.23493	0.06	0.17	8.7	3.5	even	ok

Figure S1. HRESIMS spectrum of **1**.

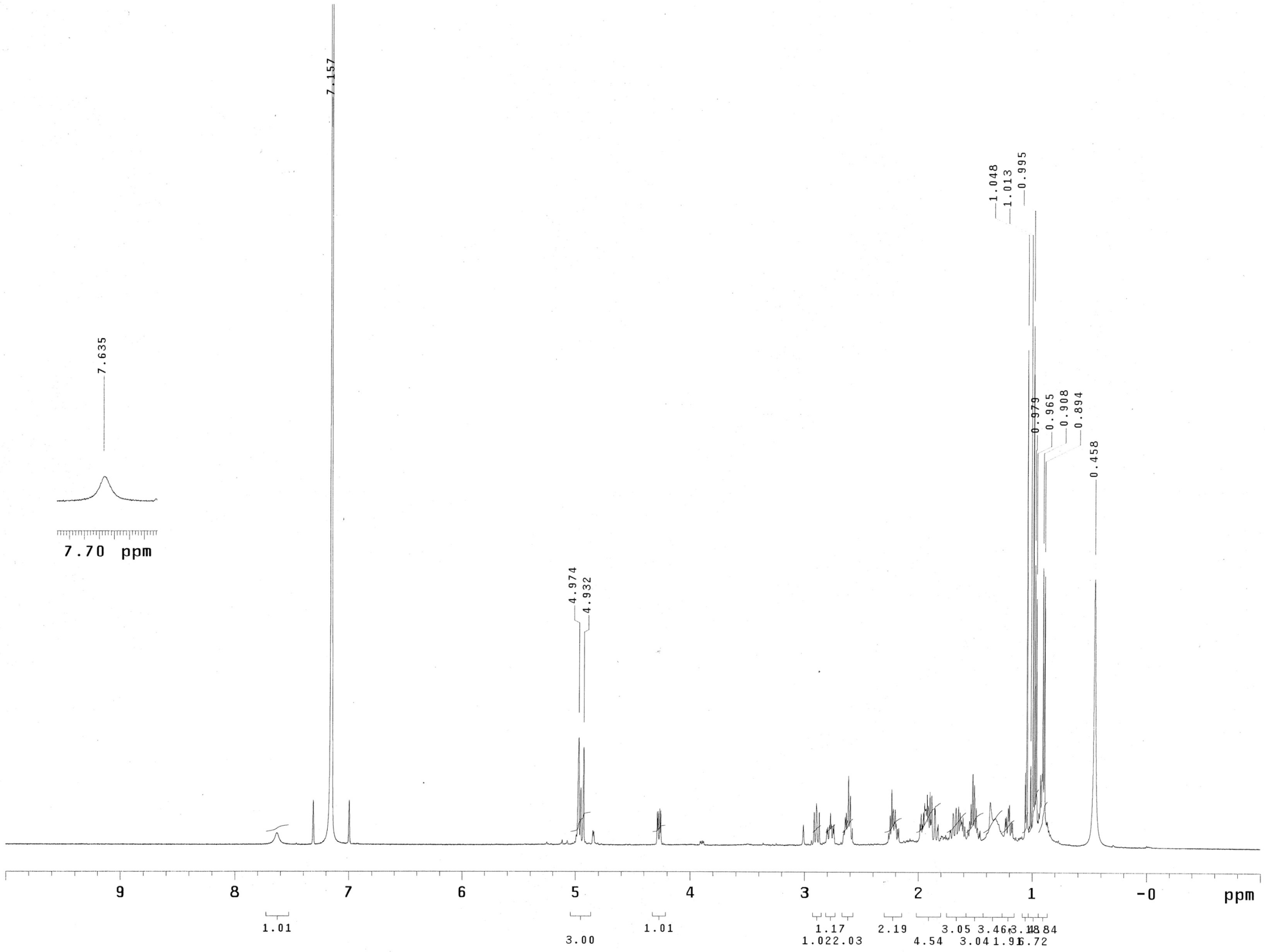

Figure S2. ^{1}H NMR spectrum of **1** in C_6D_6 at 500 MHz.

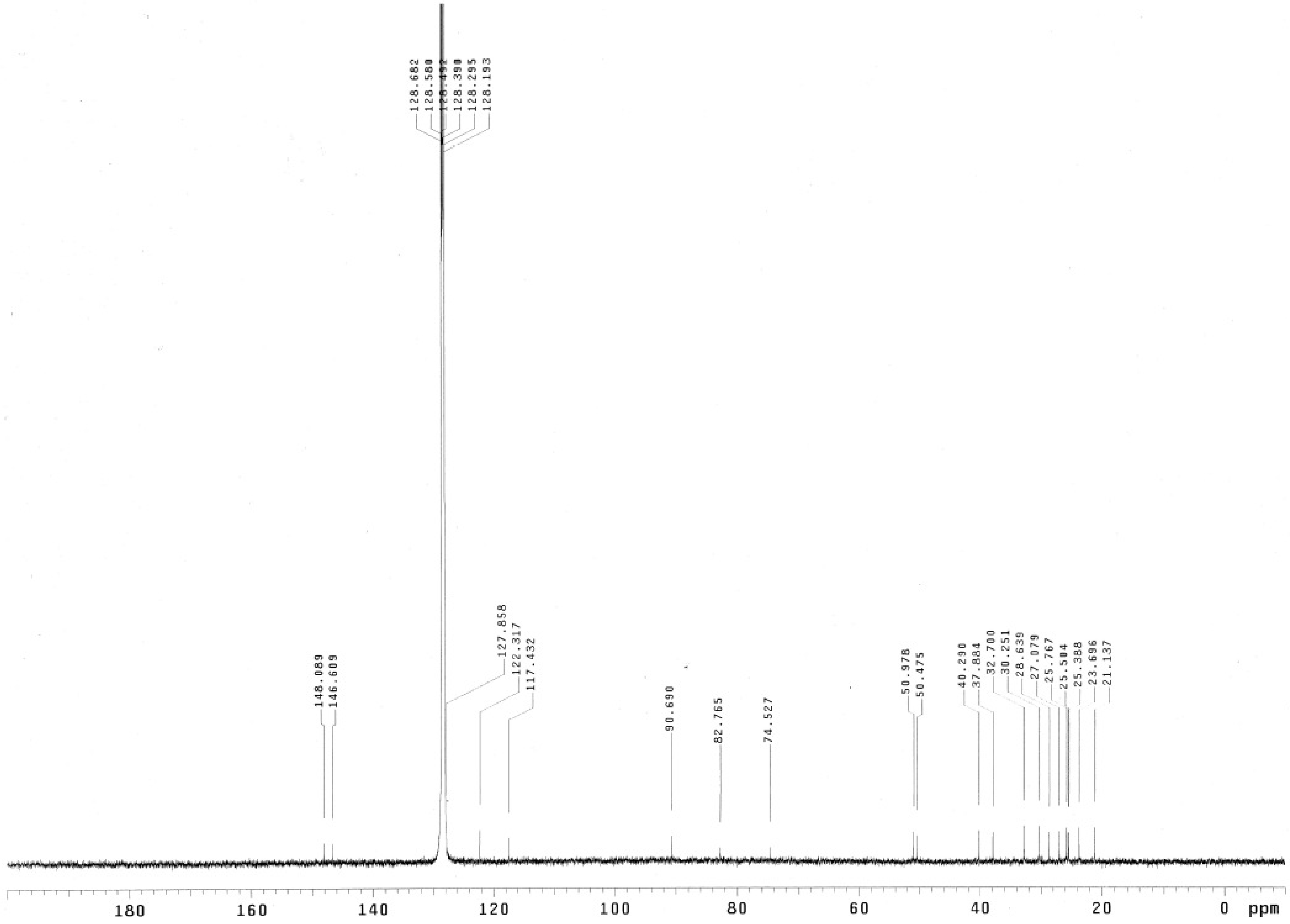

Figure S3. ^{13}C NMR spectrum of **1** in C_6D_6 at 125 MHz.

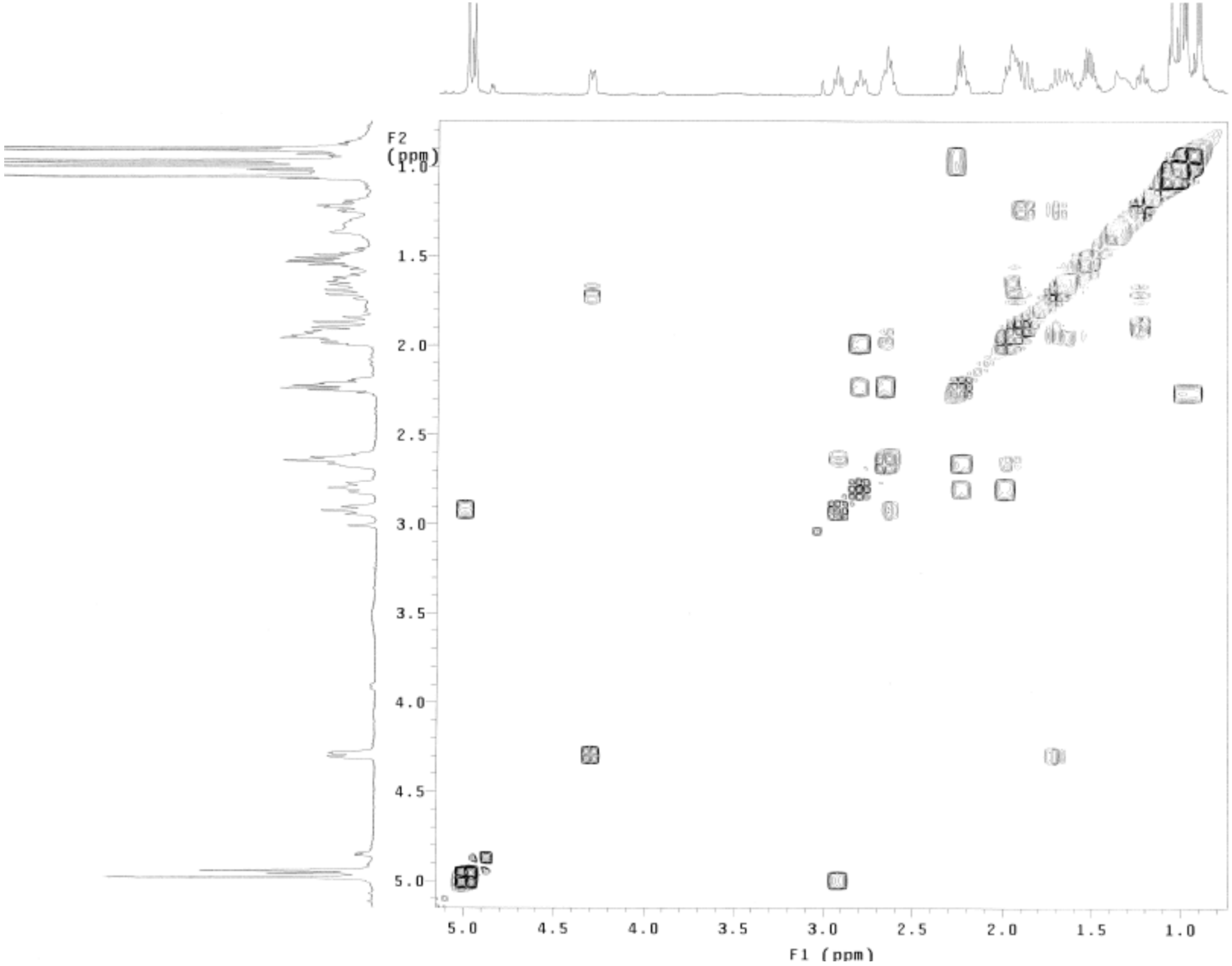

Figure S4. ^{1}H–^{1}H COSY spectrum of **1** in C_6D_6.

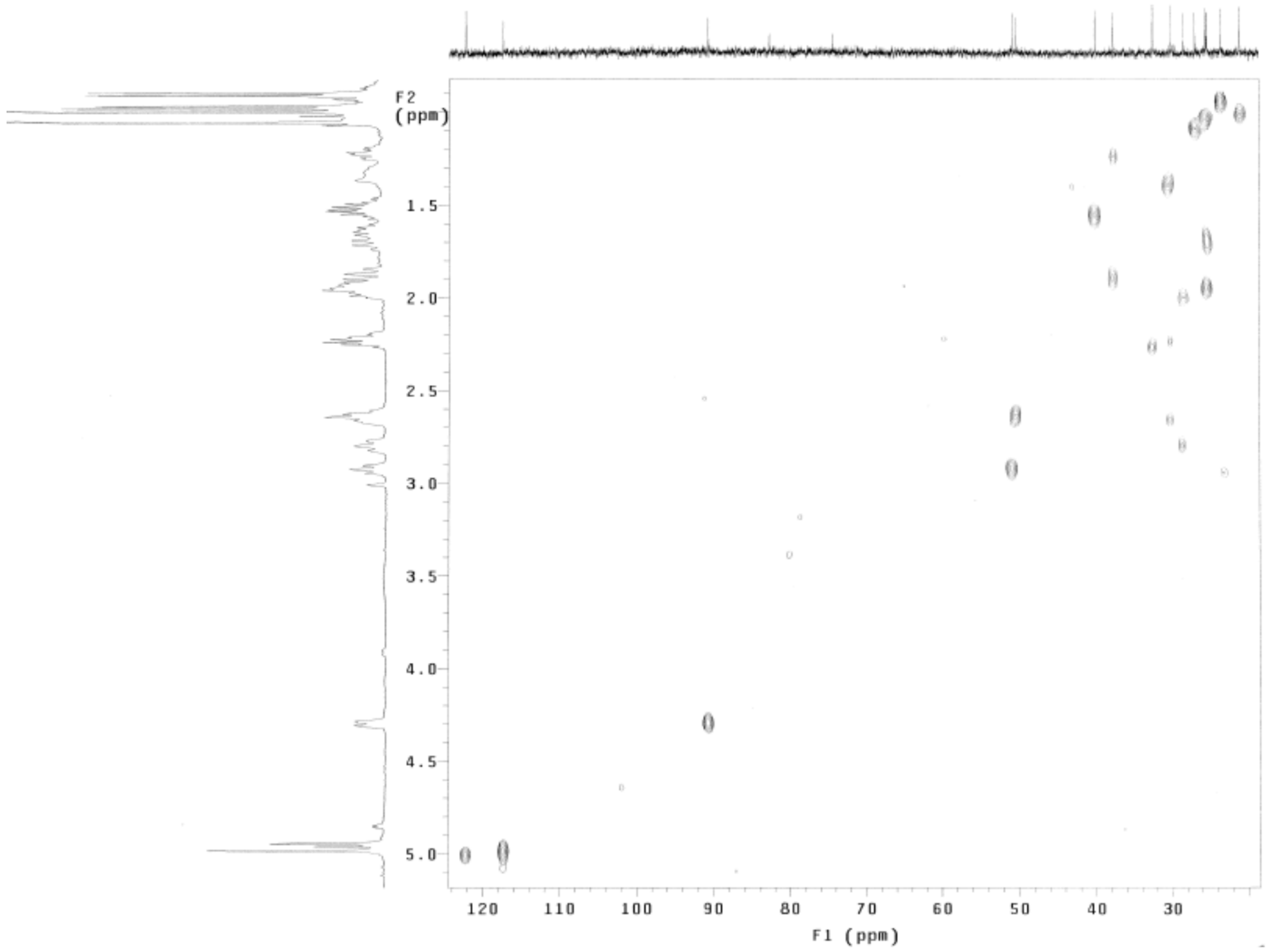

Figure S5. HSQC spectrum of **1** in C_6D_6.

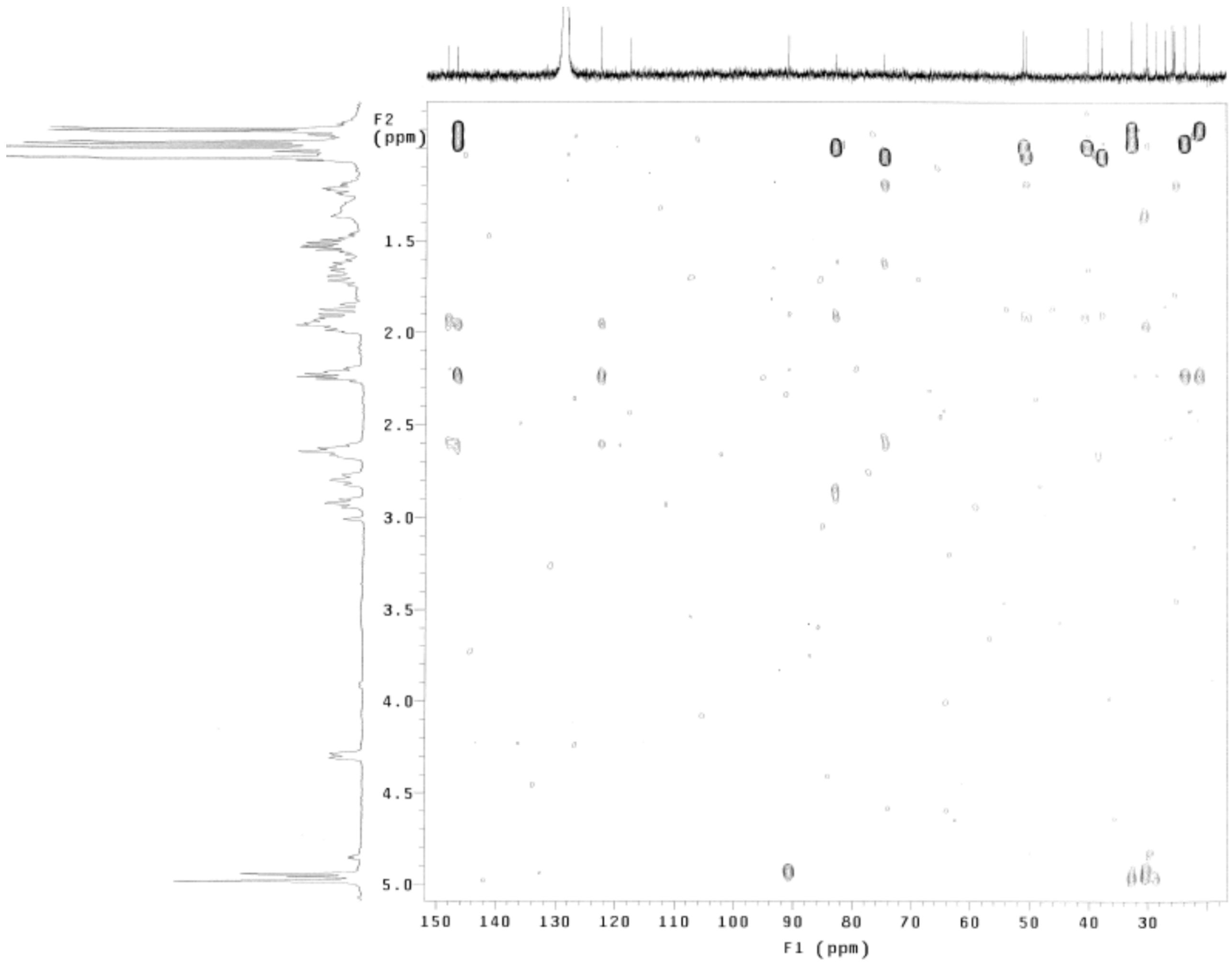

Figure S6. HMBC spectrum of **1** in C_6D_6.

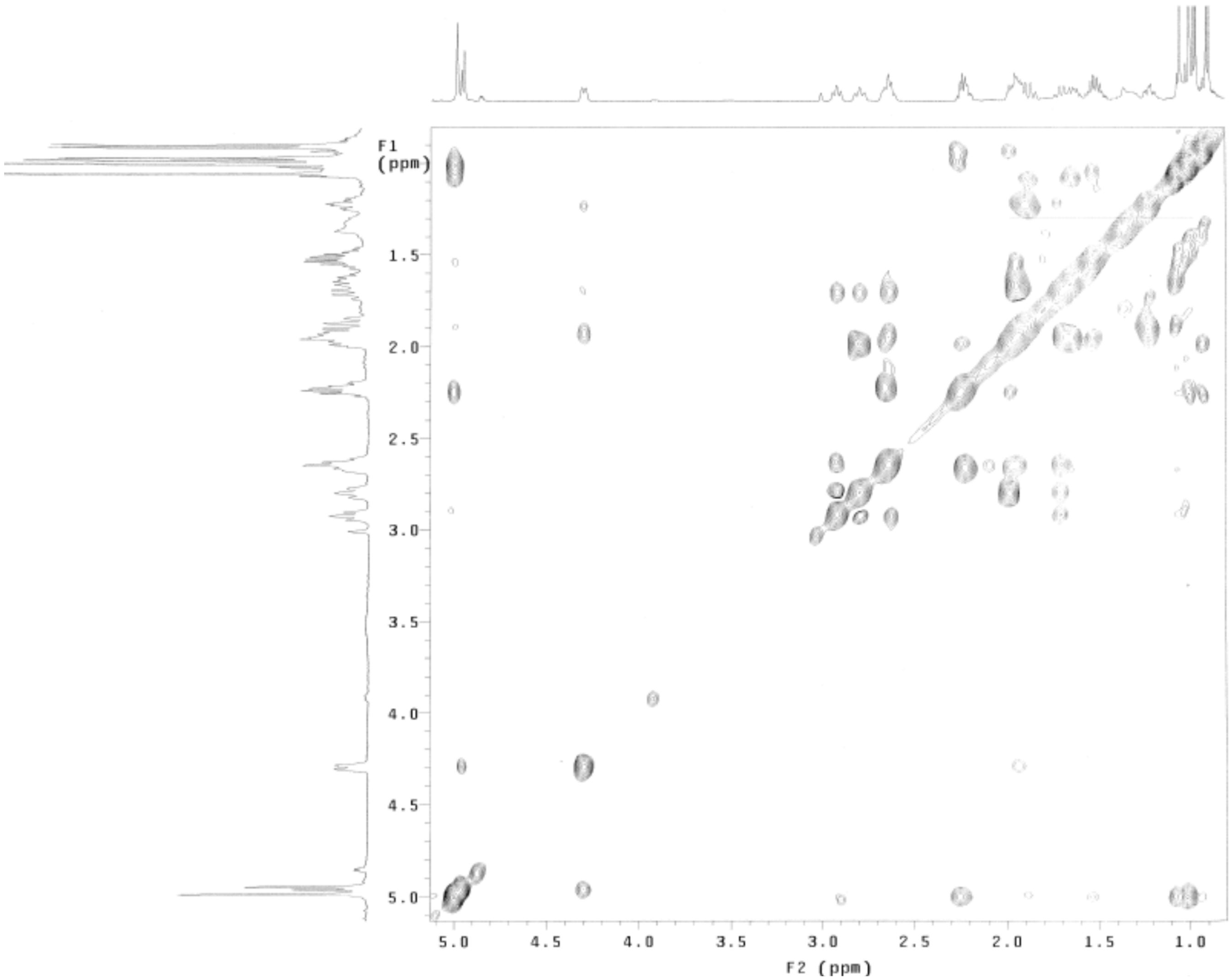

Figure S7 NOESY spectrum of **1** in C_6D_6.

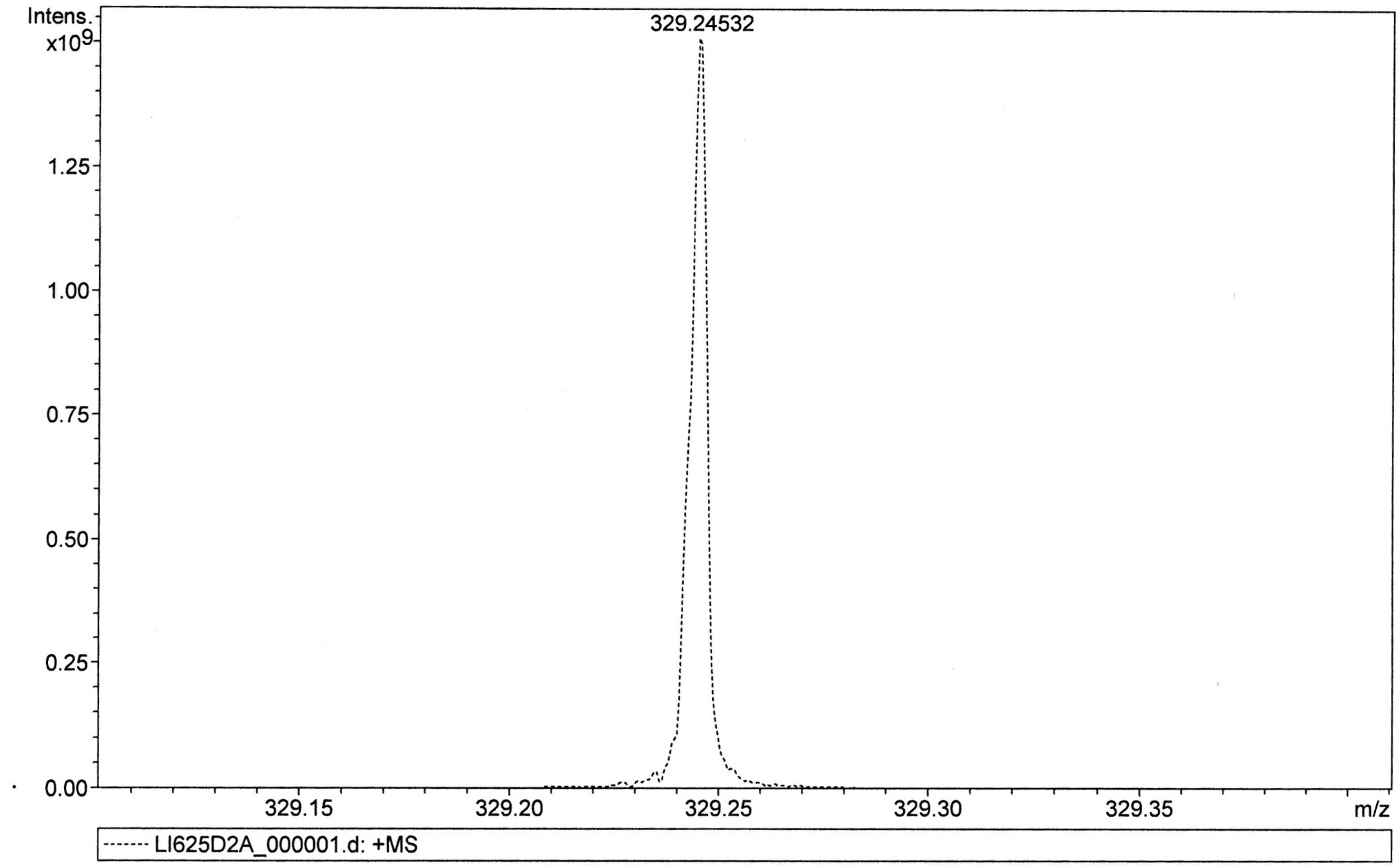

Meas. m/z	#	Formula	Score	m/z	err [mDa]	err [ppm]	mSigma	rdb	e⁻ Conf	N-Rule
329.24532	1	C 20 H 34 Na O 2	100.00	329.24510	-0.22	-0.66	3.2	3.5	even	ok

Figure S8. HRESIMS spectrum of **2**.

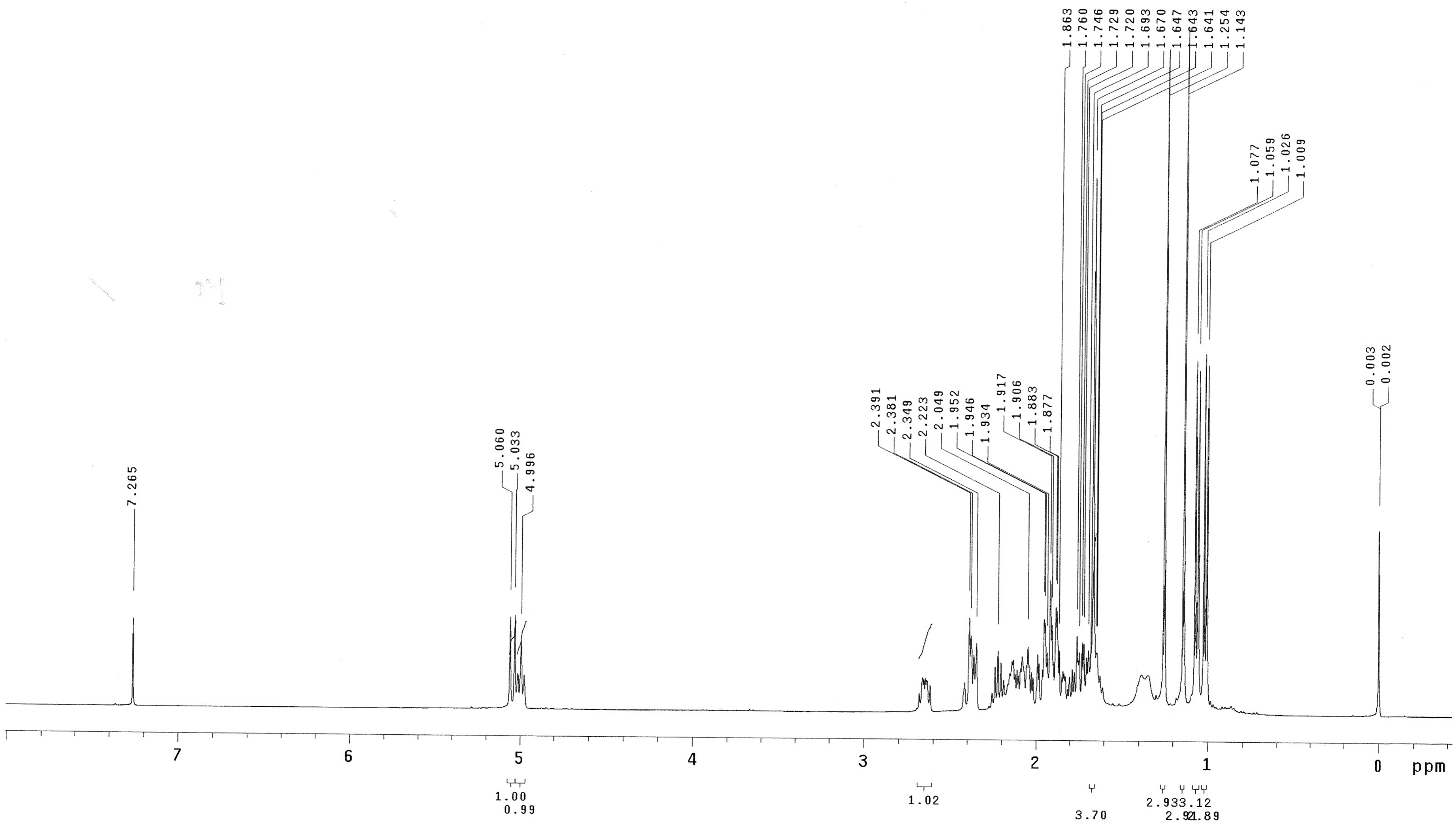

Figure S9. ^{1}H NMR spectrum of **2** in $CDCl_3$ at 400 MHz.

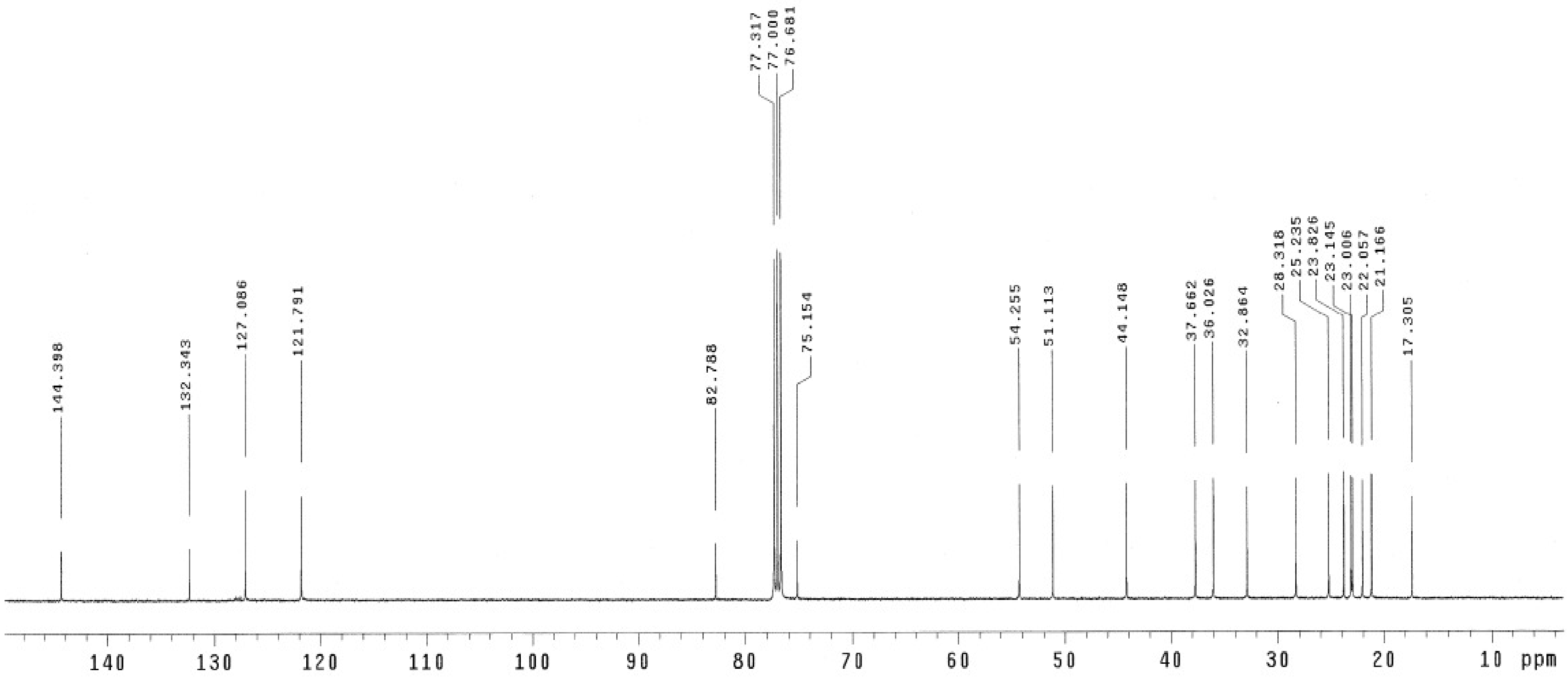

Figure S10. ^{13}C NMR spectrum of **2** in $CDCl_3$ at 100 MHz.

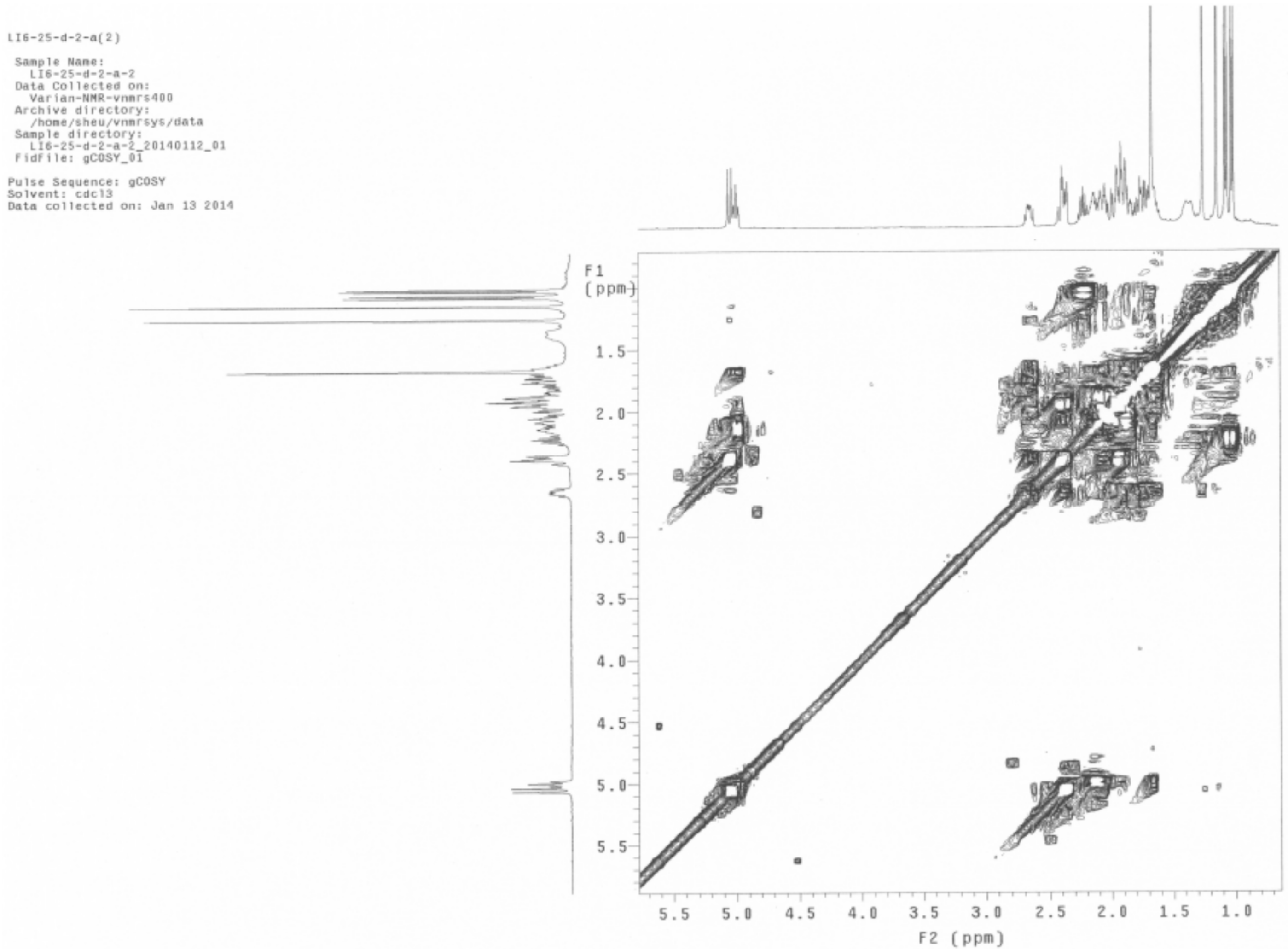

Figure S11. ^1H–^1H COSY spectrum of **2** in $CDCl_3$.

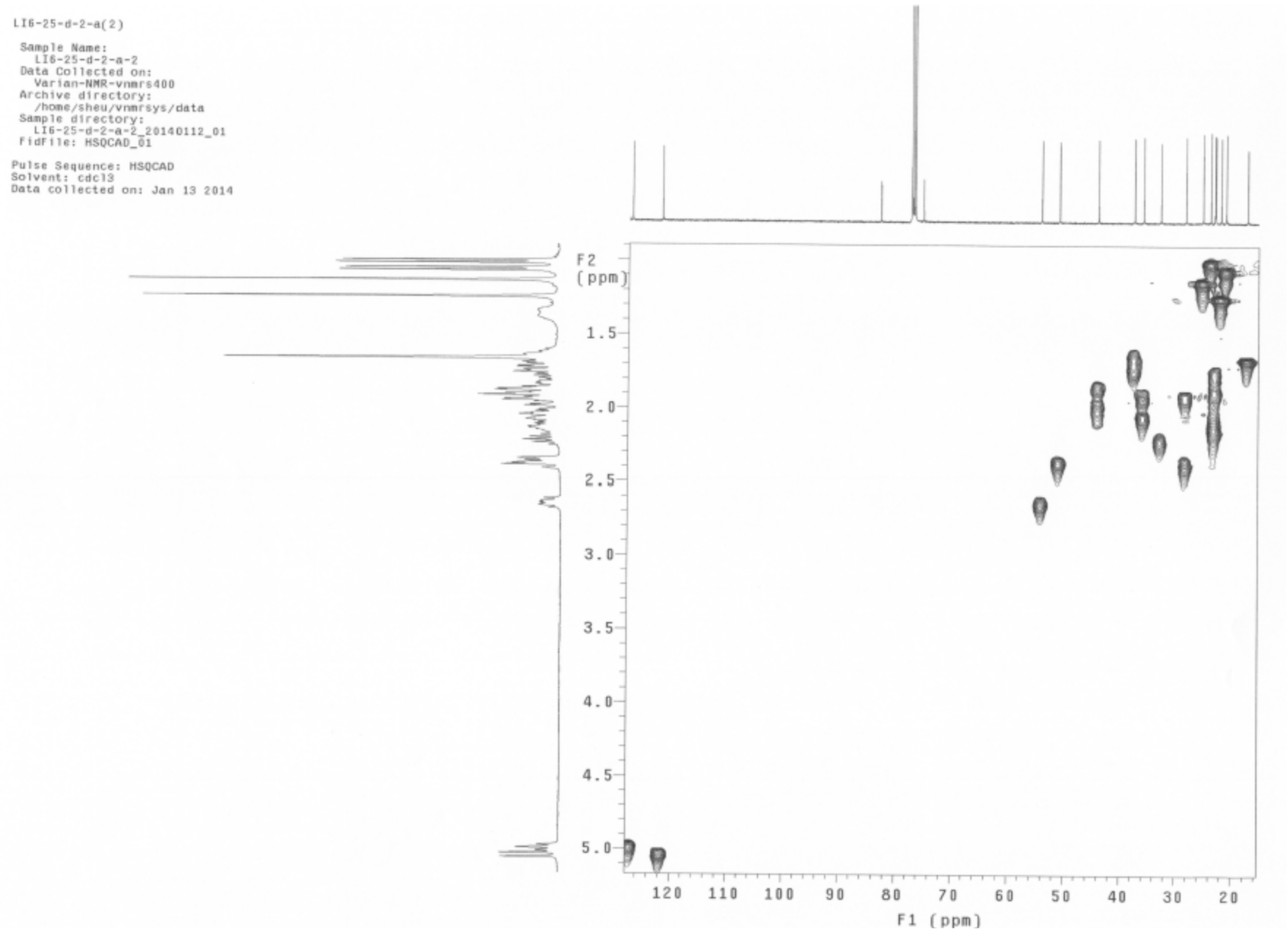

Figure S12. HSQC spectrum of **2** in $CDCl_3$.

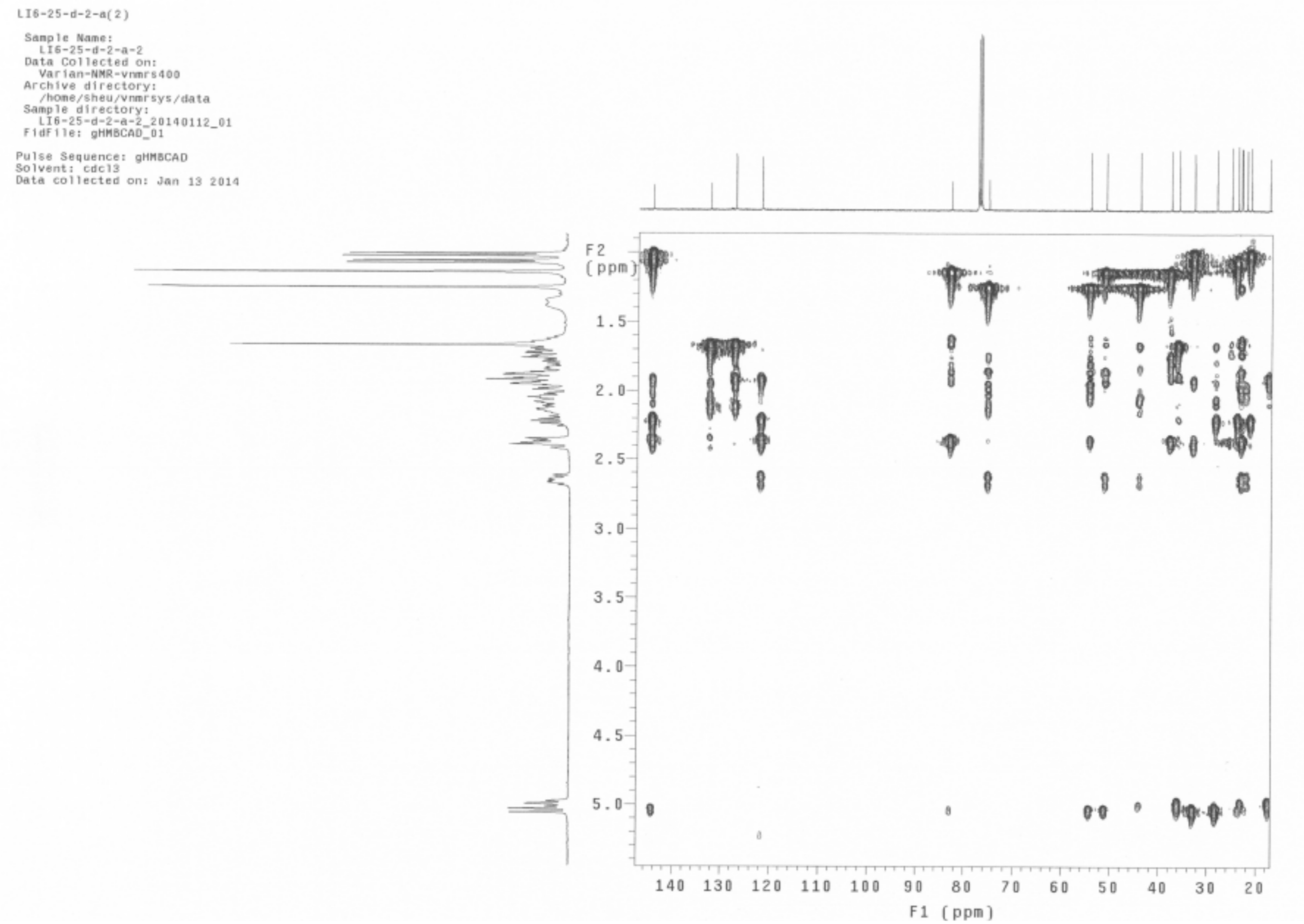

Figure S13. HMBC spectrum of **2** in $CDCl_3$.

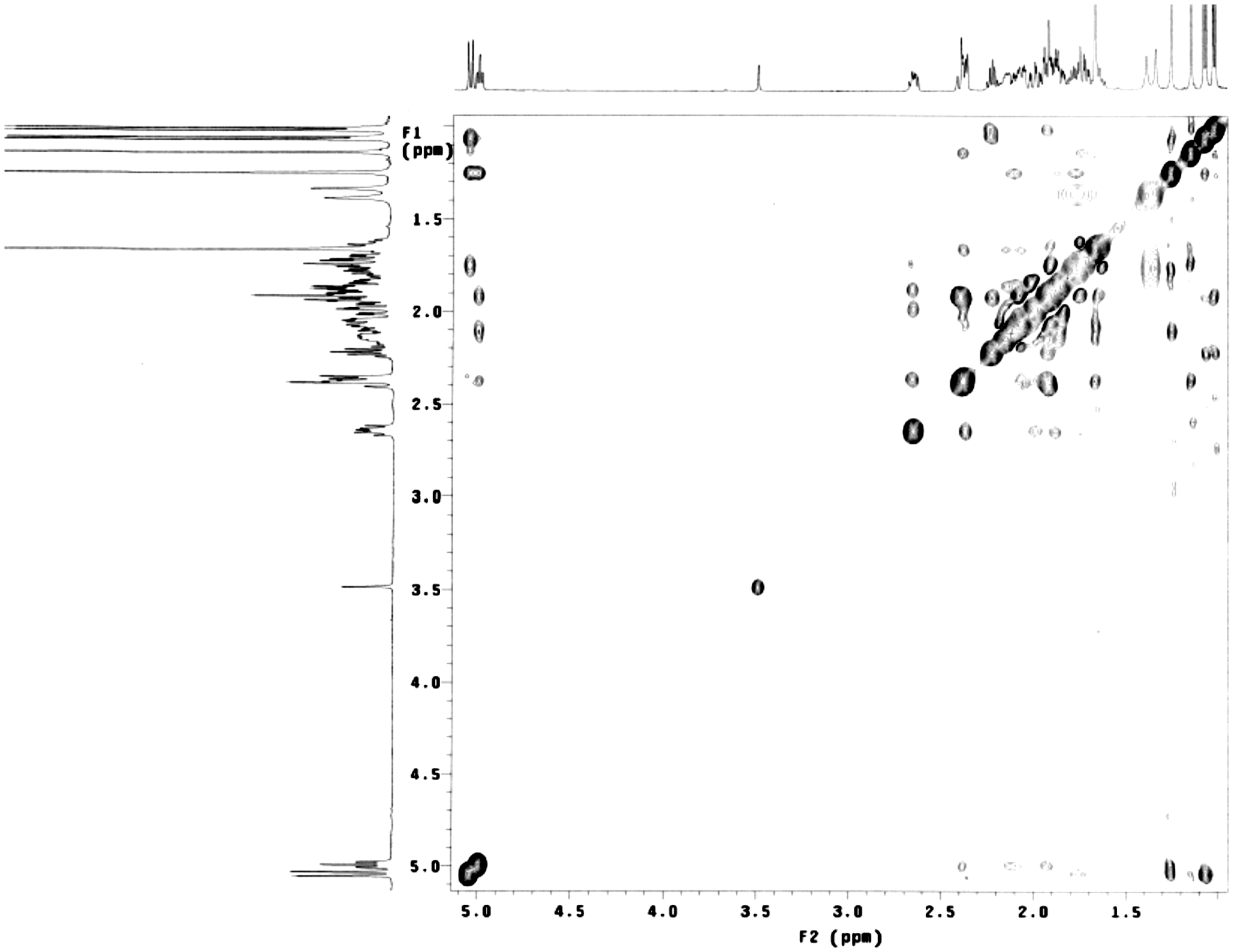

Figure S14. NOESY spectrum of **2** in $CDCl_3$.

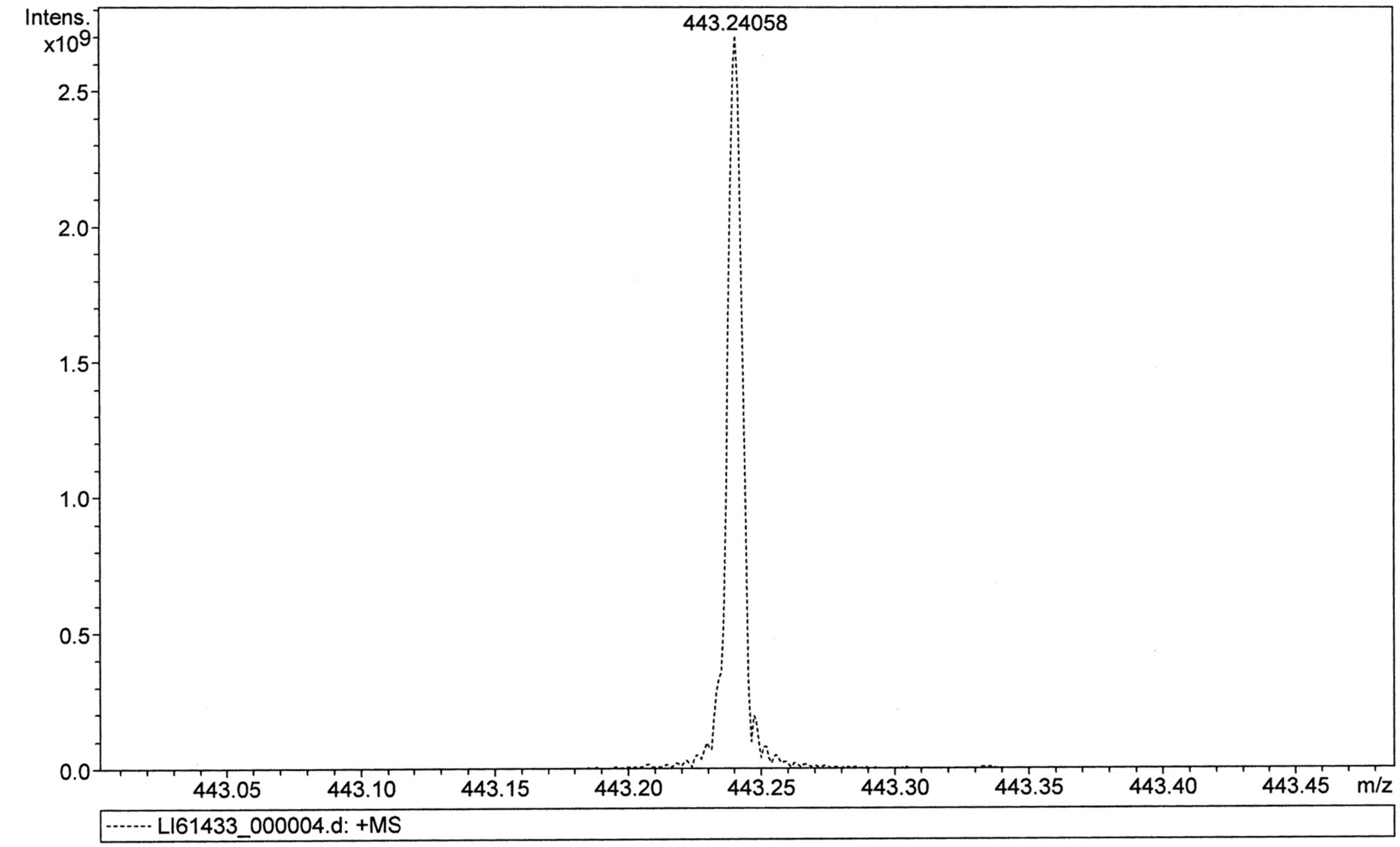

Meas. m/z	#	Formula	Score	m/z	err [mDa]	err [ppm]	mSigma	rdb	e¯ Conf	N-Rule
443.24058	1	C 24 H 36 Na O 6	100.00	443.24041	-0.17	-0.38	9.9	6.5	even	ok

Figure S15. HRESIMS spectrum of **3**.

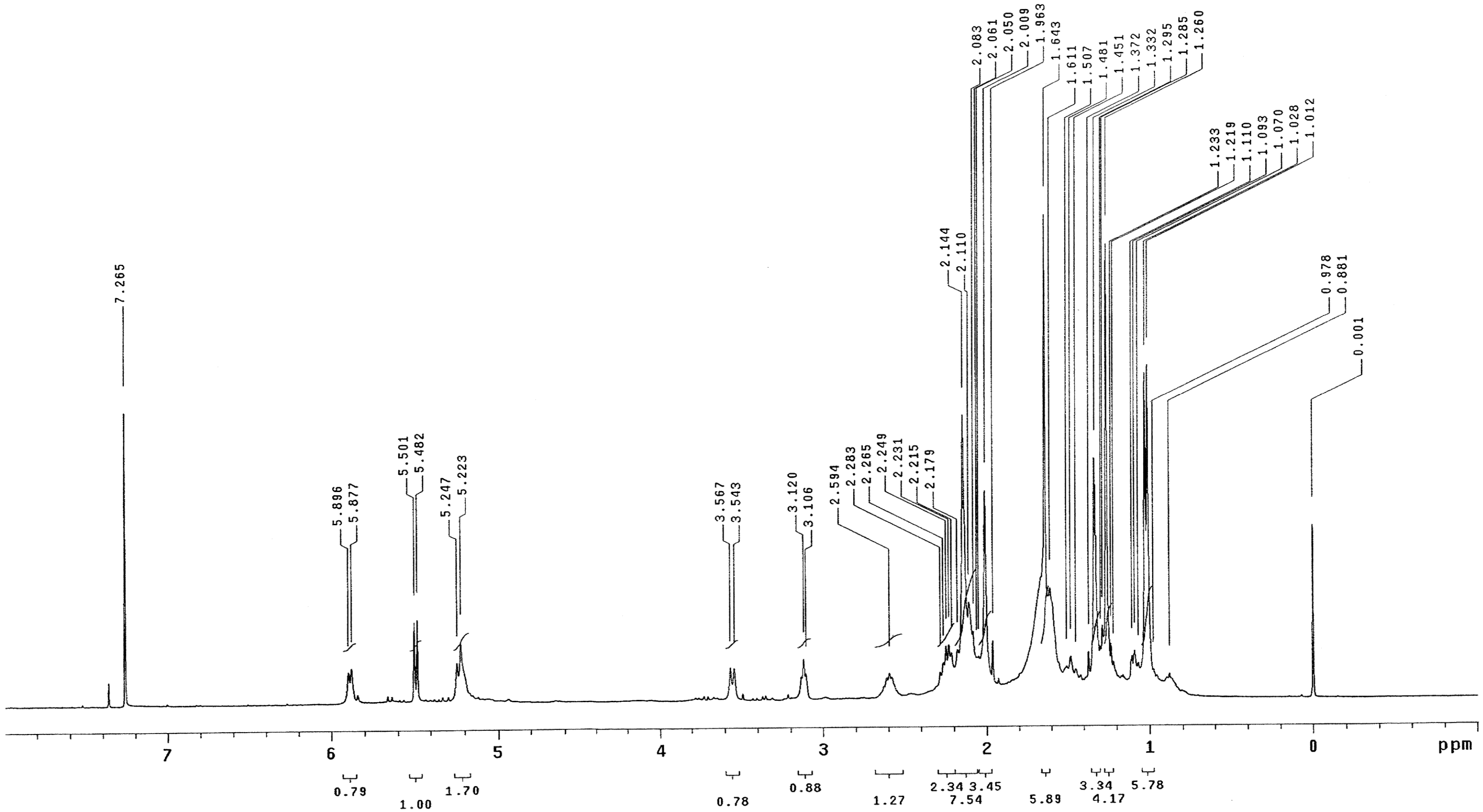

Figure S16. ^{1}H NMR spectrum of **3** in $CDCl_3$ at 400 MHz.

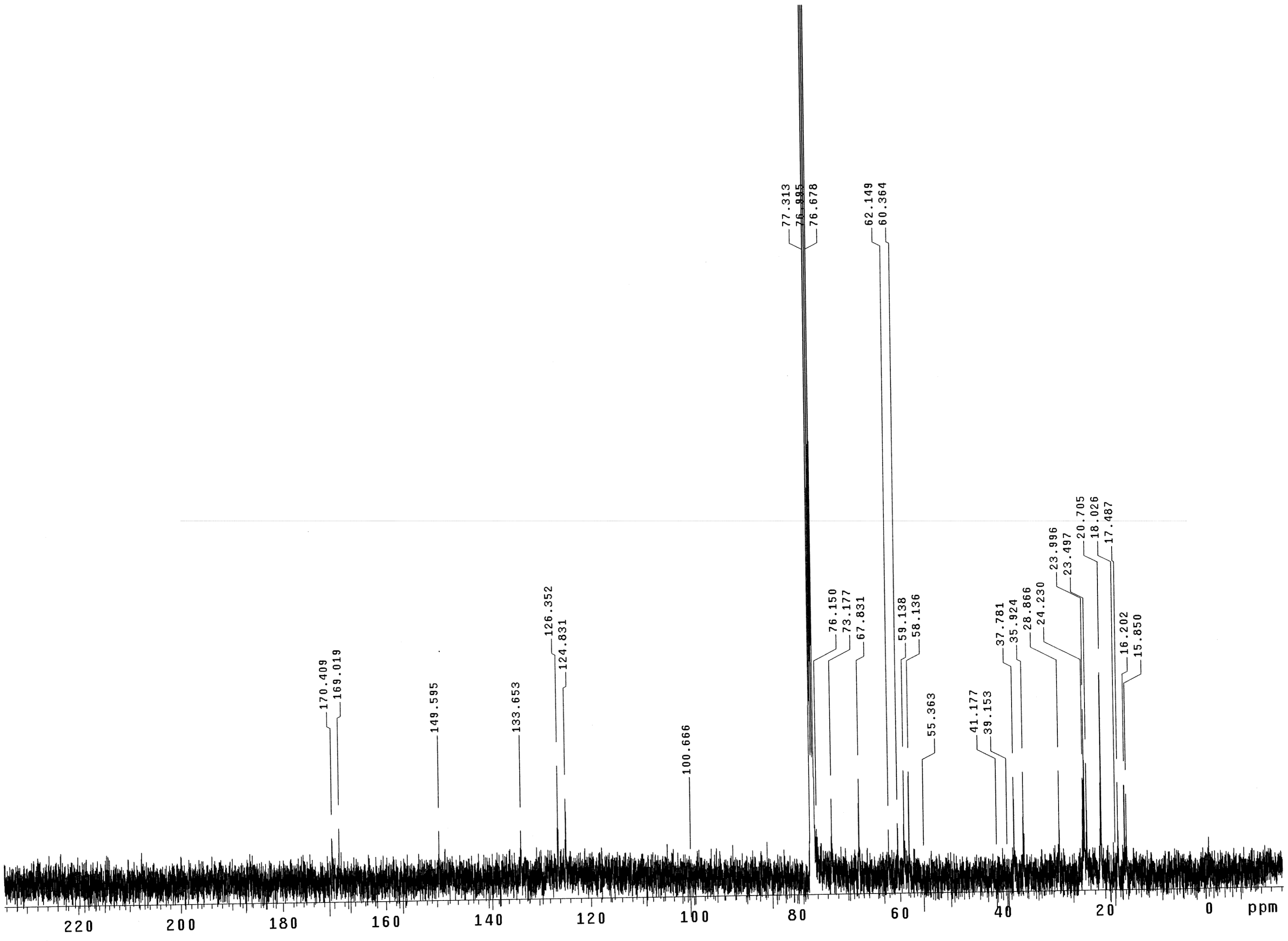

Figure S17. ^{13}C NMR spectrum of **3** in $CDCl_3$ at 100 MHz.

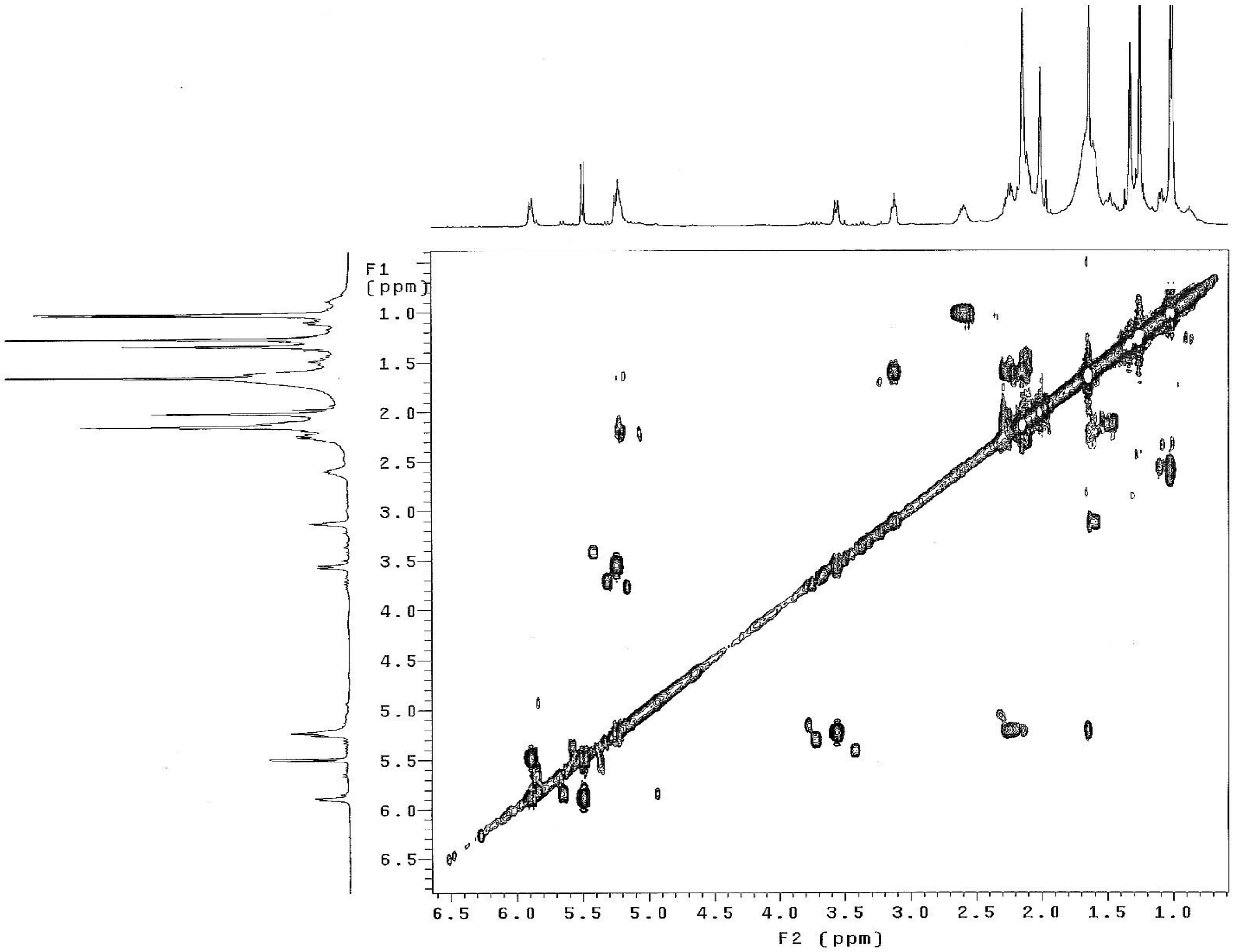

Figure S18. ^1H–^1H COSY spectrum of **3** in $CDCl_3$.

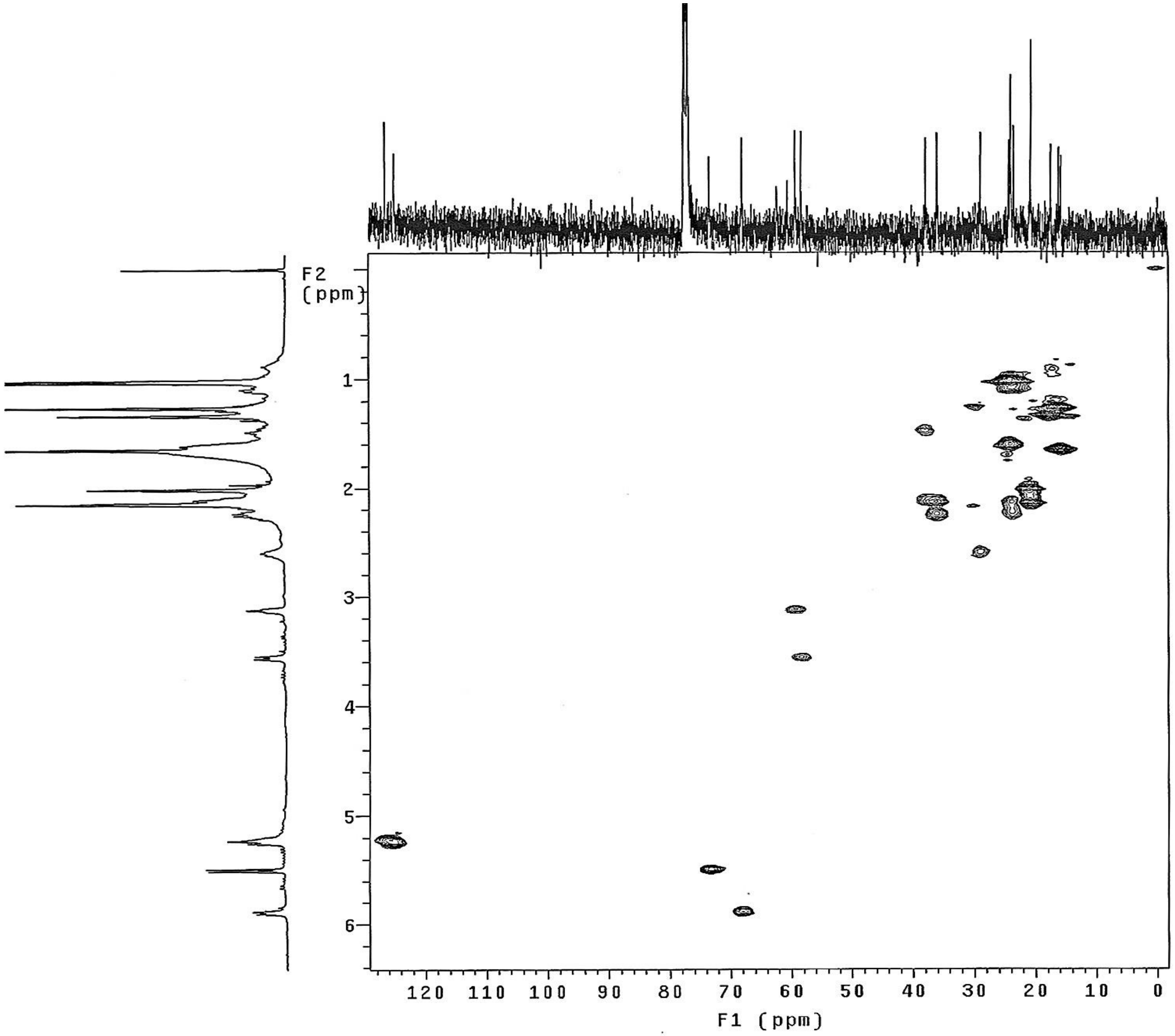

Figure S19. HSQC spectrum of **3** in $CDCl_3$.

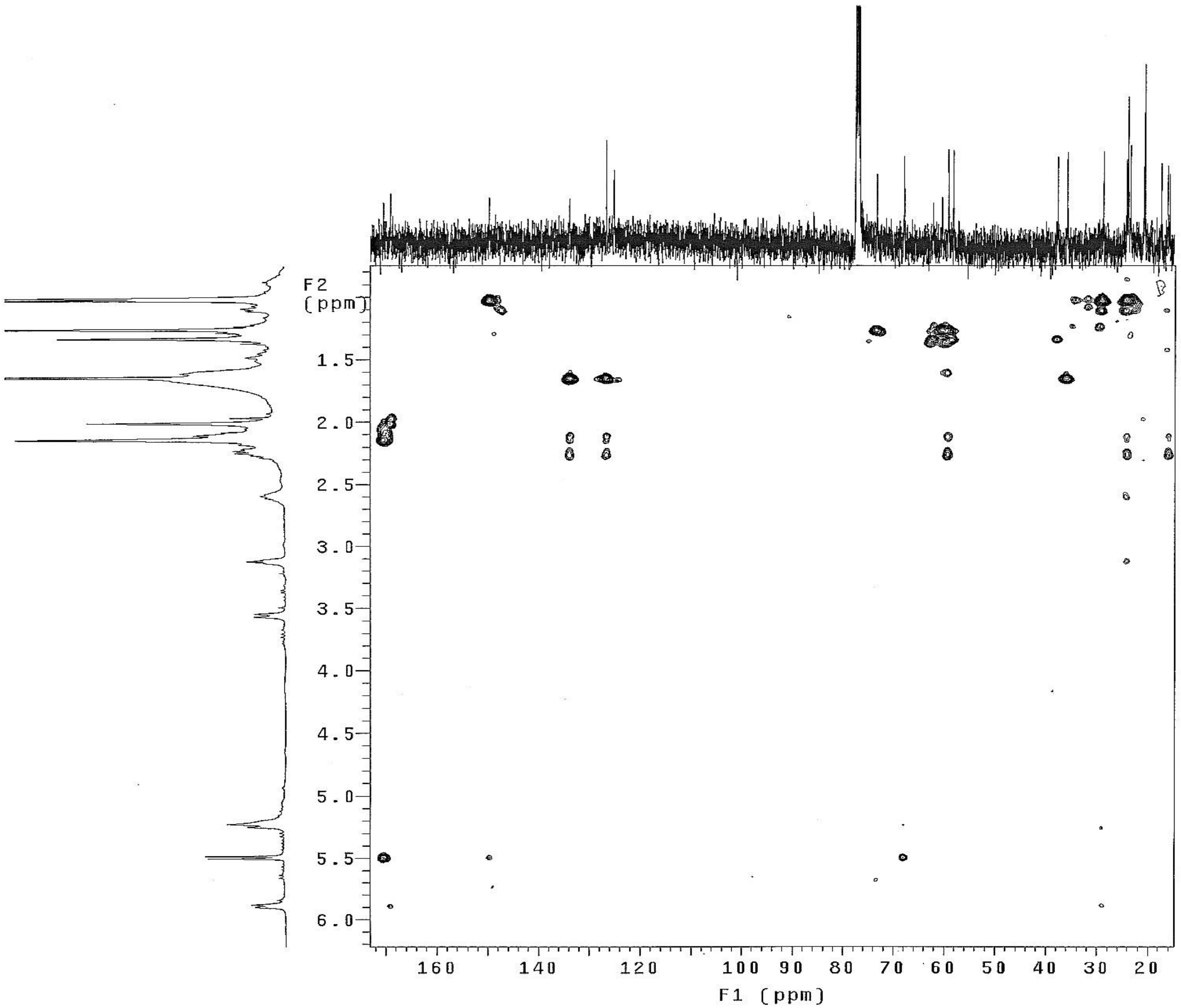

Figure S20. HMBC spectrum of **3** in $CDCl_3$.

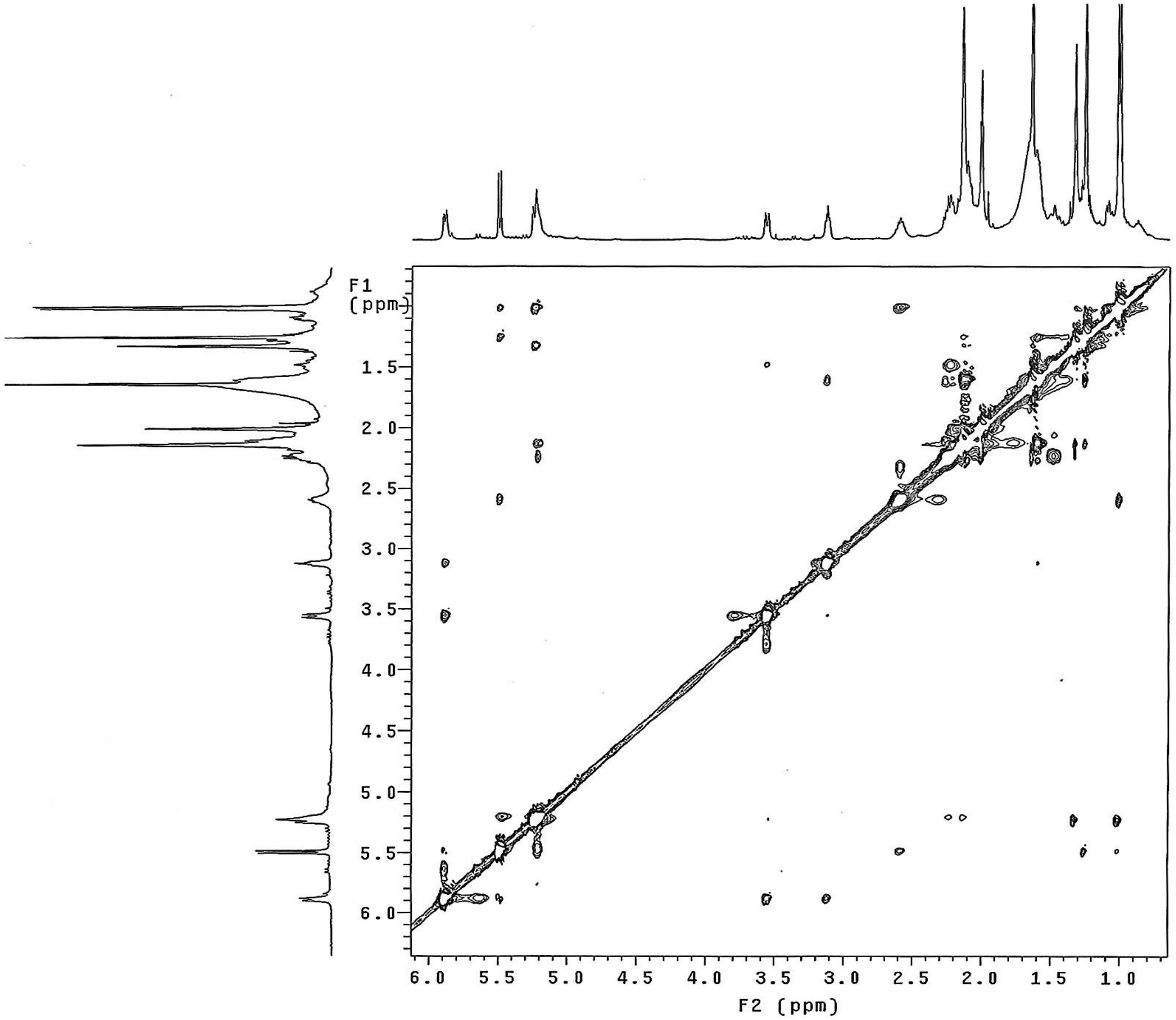

Figure S21. NOESY spectrum of **3** in $CDCl_3$.

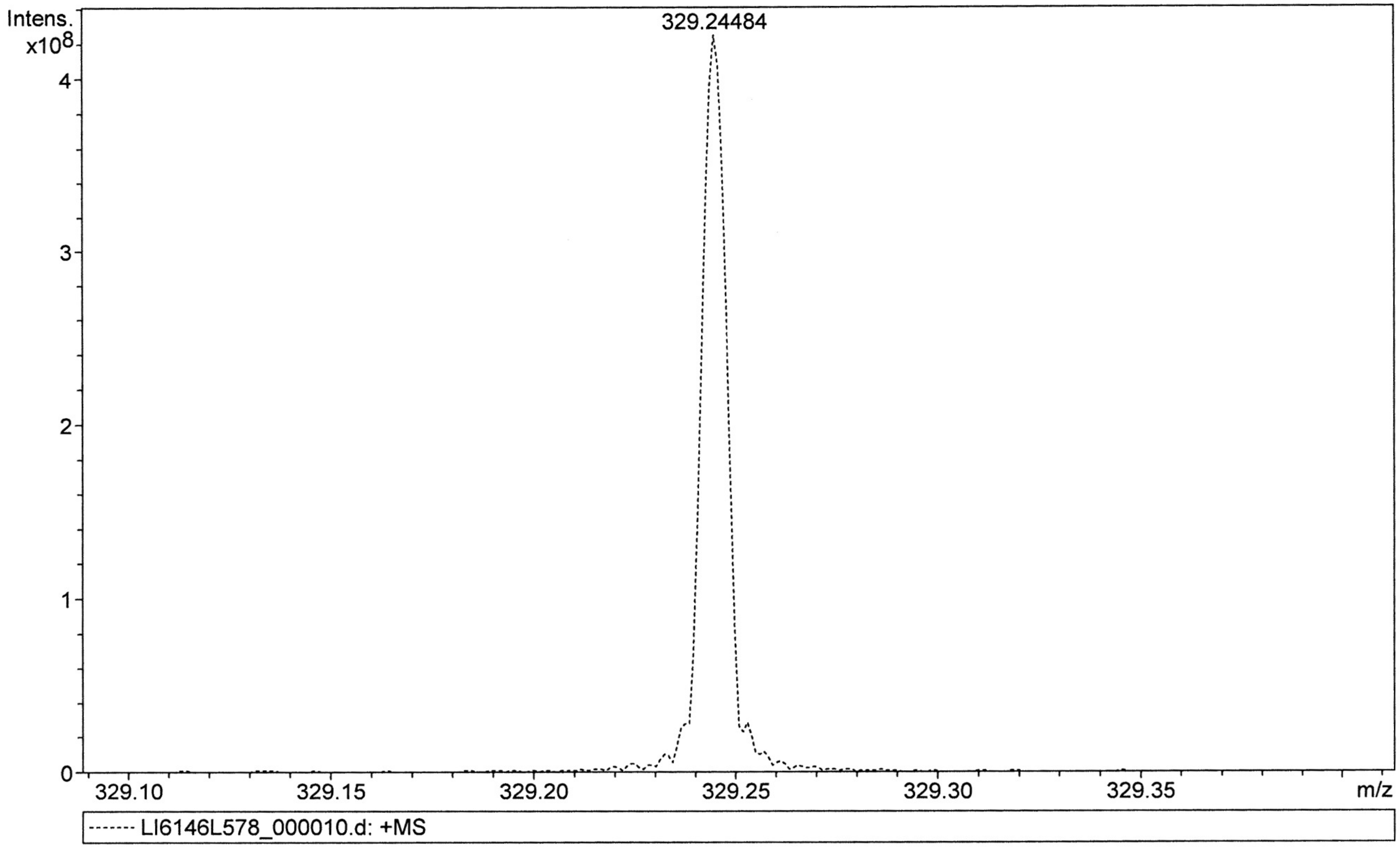

Meas. m/z	#	Formula	Score	m/z	err [mDa]	err [ppm]	mSigma	rdb	e⁻ Conf	N-Rule
329.24484	1	C 20 H 34 Na O 2	100.00	329.24510	0.27	0.81	3.6	3.5	even	ok

Figure S22. HRESIMS spectrum of **4**.

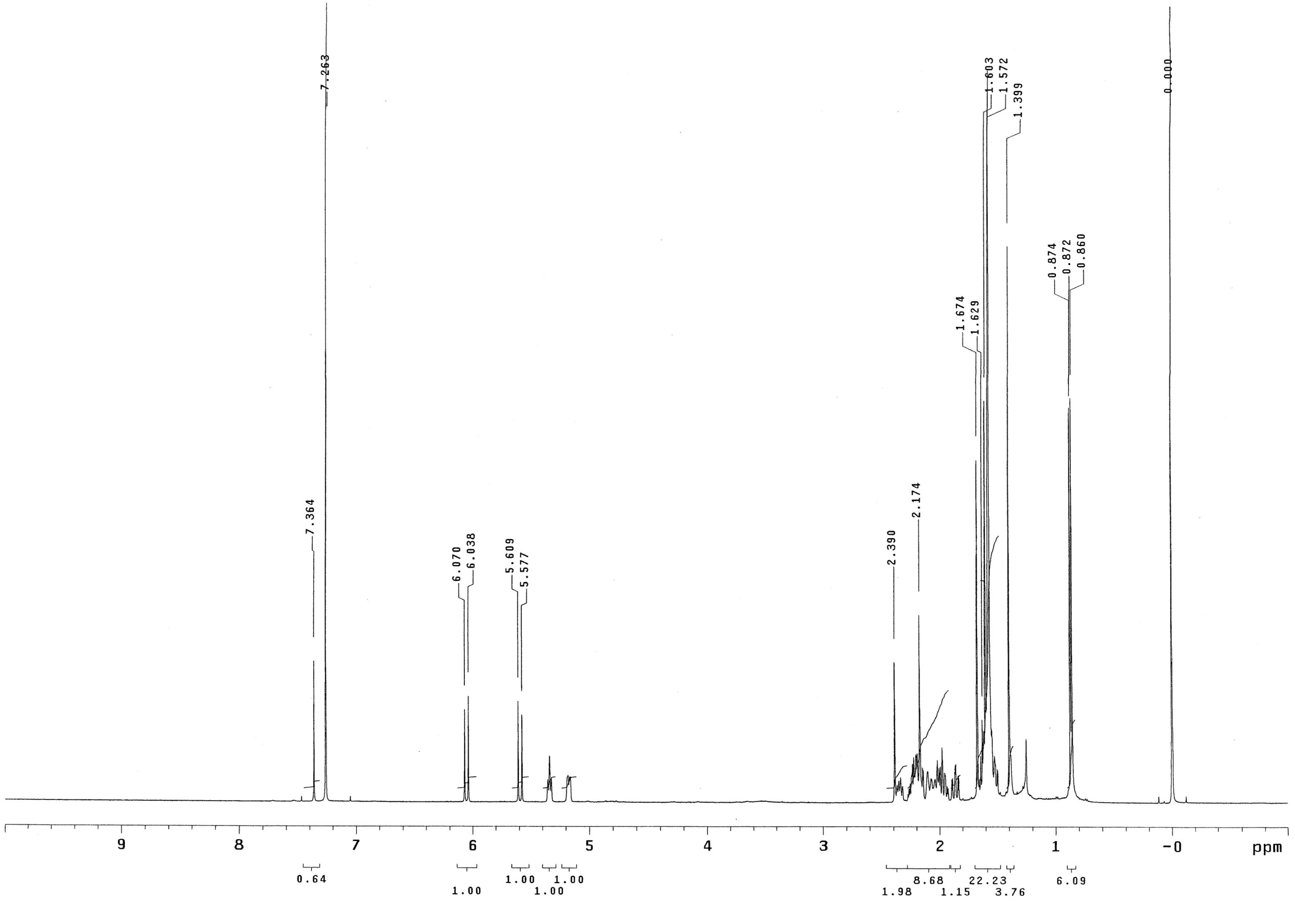

Figure S23. ^{1}H NMR spectrum of **4** in $CDCl_3$ at 500 MHz.

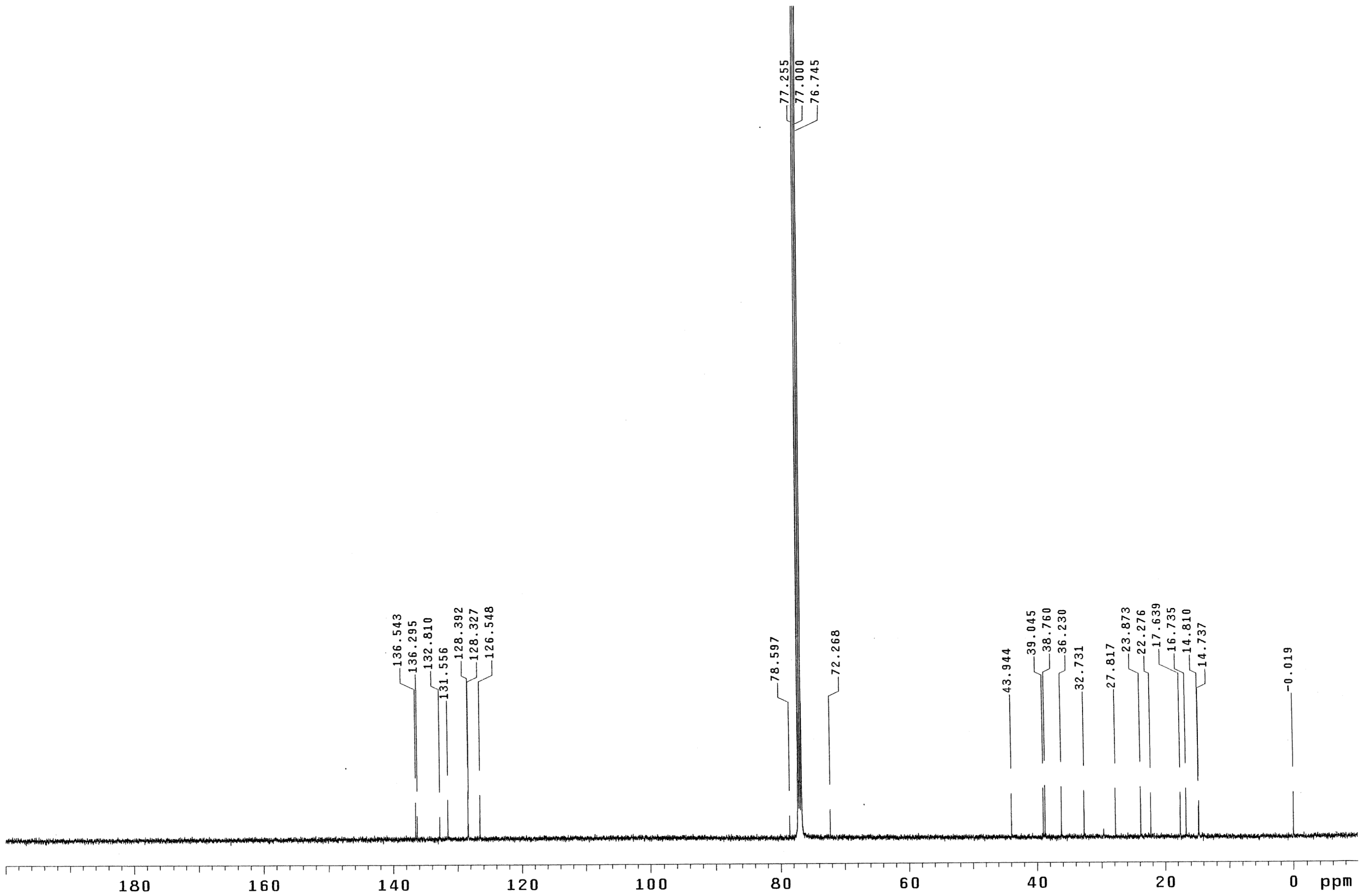

Figure S24. ^{13}C NMR spectrum of **4** in $CDCl_3$ at 125 MHz.

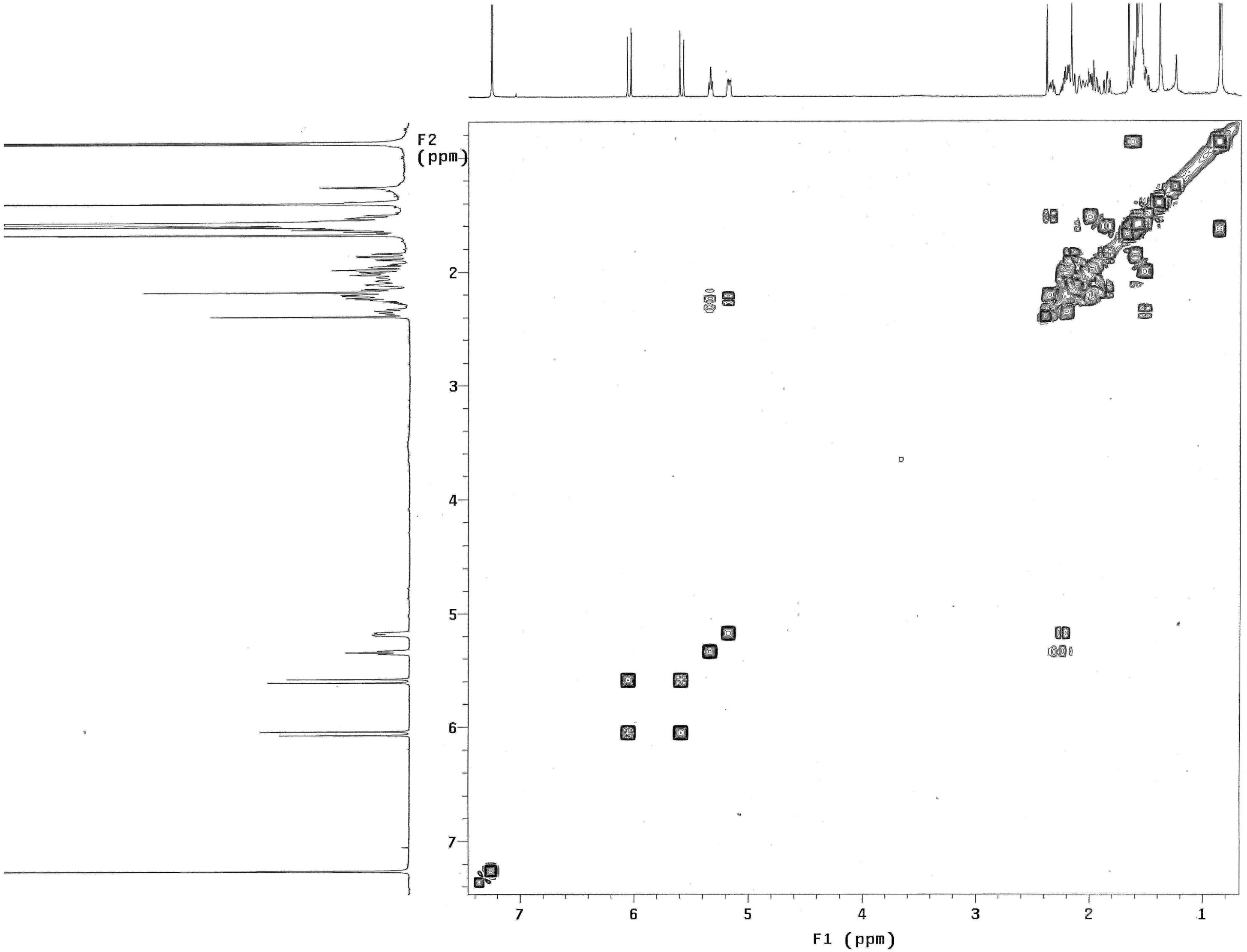

Figure S25. ^{1}H–^{1}H COSY spectrum of **4** in $CDCl_3$.

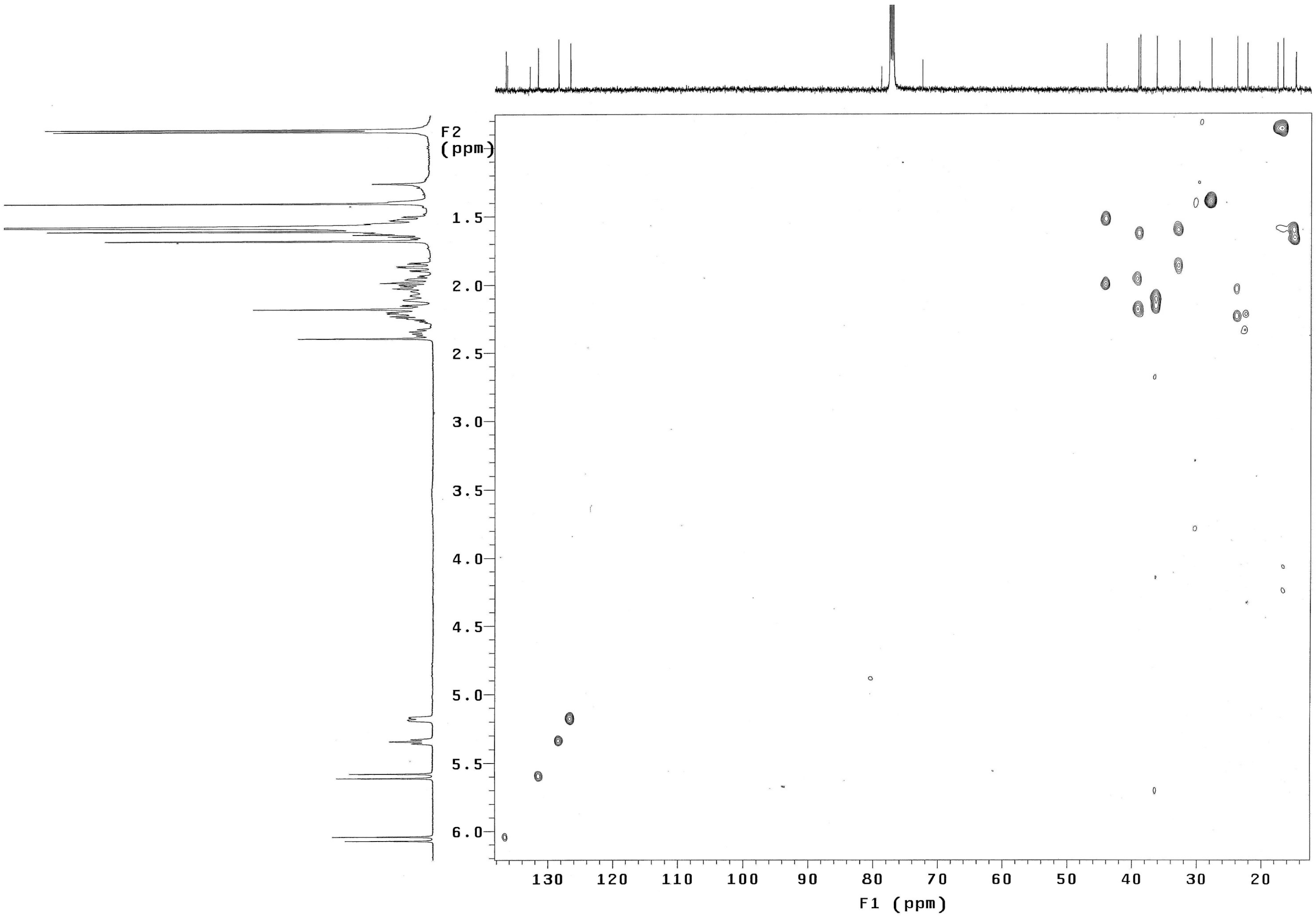

Figure S26. HSQC spectrum of **4** in $CDCl_3$.

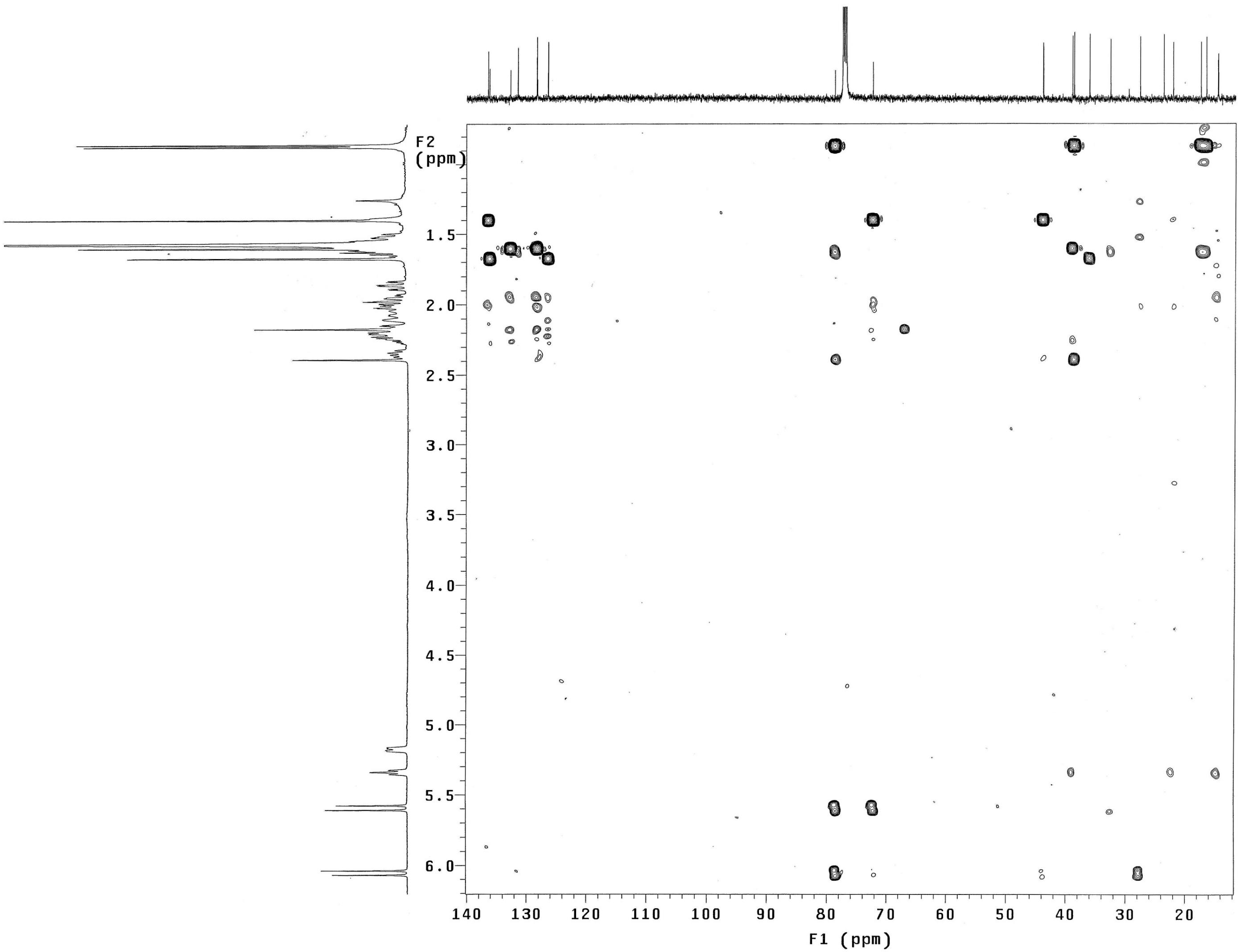

Figure S27. HMBC spectrum of **4** in $CDCl_3$.

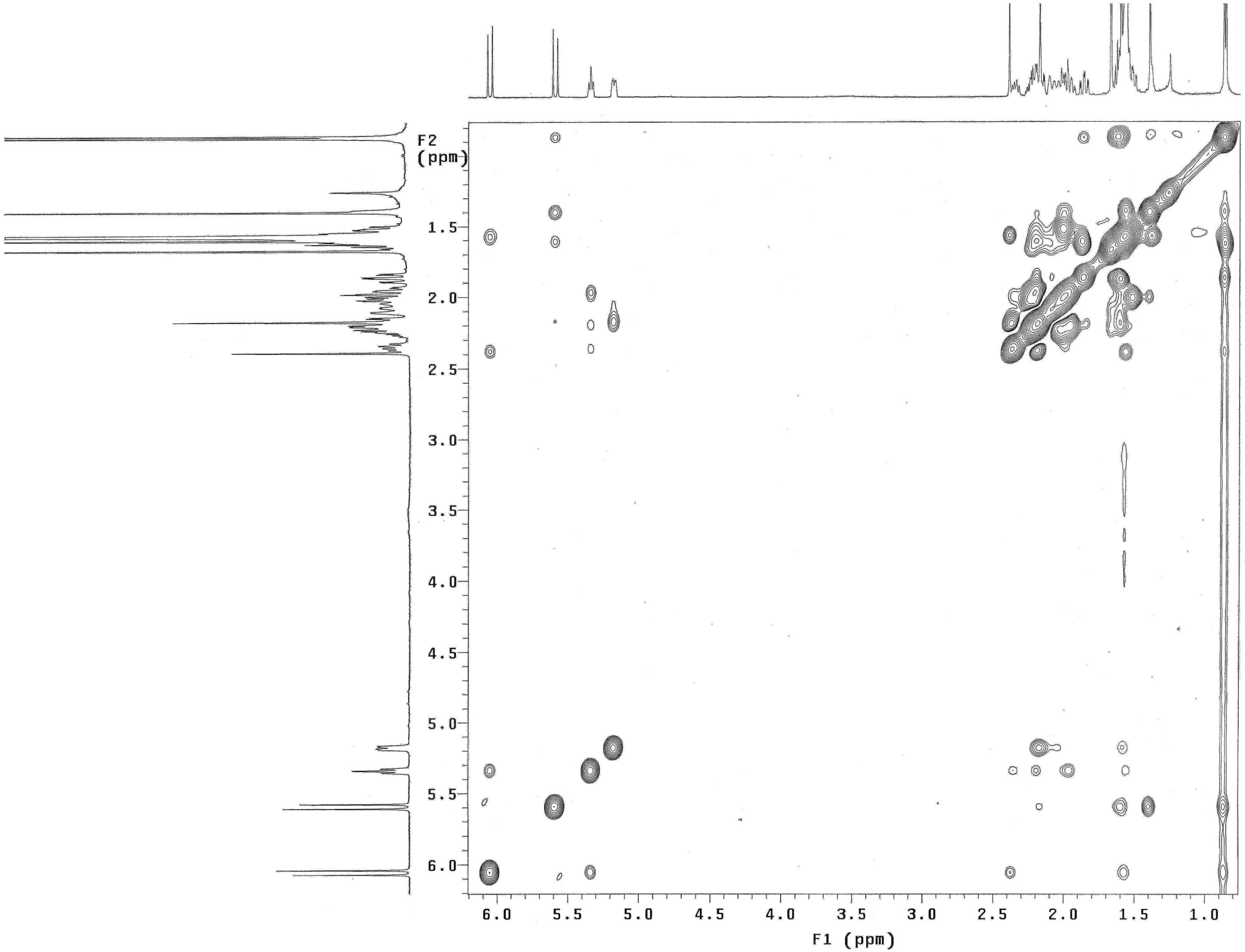

Figure S28. NOESY spectrum of **4** in $CDCl_3$.
